# Interleukin-31 promotes fibrosis and T helper 2 polarization in systemic sclerosis

**DOI:** 10.1038/s41467-021-26099-w

**Published:** 2021-10-12

**Authors:** Ai Kuzumi, Ayumi Yoshizaki, Kazuki M. Matsuda, Hirohito Kotani, Yuta Norimatsu, Maiko Fukayama, Satoshi Ebata, Takemichi Fukasawa, Asako Yoshizaki-Ogawa, Yoshihide Asano, Kyojiro Morikawa, Yutaka Kazoe, Kazuma Mawatari, Takehiko Kitamori, Shinichi Sato

**Affiliations:** 1 Department of Dermatology, Graduate School of Medicine, The University of Tokyo, Tokyo, Japan; 2 Department of Applied Chemistry, Graduate School of Engineering, The University of Tokyo, Tokyo, Japan; 3 Department of System Design Engineering, Faculty of Science and Technology, Keio University, Yokohama, Japan; 4 Department of Bioengineering, Graduate School of Engineering, The University of Tokyo, Tokyo, Japan

**Keywords:** Autoimmunity, Interleukins, T-helper 2 cells, Rheumatology

## Abstract

Systemic sclerosis (SSc) is a chronic multisystem disorder characterized by fibrosis and autoimmunity. Interleukin (IL)-31 has been implicated in fibrosis and T helper (Th) 2 immune responses, both of which are characteristics of SSc. The exact role of IL-31 in SSc pathogenesis is unclear. Here we show the overexpression of IL-31 and IL-31 receptor A (IL-31RA) in dermal fibroblasts (DFs) from SSc patients. We elucidate the dual role of IL-31 in SSc, where IL-31 directly promotes collagen production in DFs and indirectly enhances Th2 immune responses by increasing pro-Th2 cytokine expression in DFs. Furthermore, blockade of IL-31 with anti-IL-31RA antibody significantly ameliorates fibrosis and Th2 polarization in a mouse model of SSc. Therefore, in addition to defining IL-31 as a mediator of fibrosis and Th2 immune responses in SSc, our study provides a rationale for targeting the IL-31/IL-31RA axis in the treatment of SSc.

## Abstract (web summary)

Systemic sclerosis (SSc) disease involves multisystem fibrosis and autoimmunity with limited treatment options. Here the authors demonstrate that IL-31 and IL-31RA are overexpressed in dermal fibroblasts from SSc patients and show that fibrosis and cytokine release can be reduced upon blocking of IL-31/IL-31RA.

## Introduction

Systemic sclerosis (SSc) is a connective tissue disease characterized by excessive extracellular matrix deposition of the skin and internal organs^1,2^. The consequent fibrosis leads to tissue dysfunction and organ failure that can be debilitating and life threatening. Although the pathogenesis of SSc still remains unknown, various immunological abnormalities have been reported in SSc patients, indicating the autoimmune nature of the disease. In particular, activation and polarization of T cells have been extensively studied both in patients and in animal models of SSc^3^. For example, CD4^+^ T cells have been shown to infiltrate the lesional skin during the early stage of SSc^4^. These tissue-infiltrating T cells show increased expression of activation markers^5^. T cells in the peripheral blood have also been found to be activated in SSc^6^. In addition, activated CD4^+^ T cells in SSc are predominantly skewed to T helper (Th) 2, which is implicated in tissue fibrosis^3,7^. Indeed, major Th2 cytokines such as interleukin (IL)-4 and IL-13 are overexpressed in the skin and serum of SSc patients^8,9^. Th2 cytokines are also associated with skin and lung fibrosis in bleomycin-induced SSc model (BLM-SSc) mice, a well-established experimental model of SSc^10,11^. Mechanistically, IL-4 and IL-13 directly induce collagen production in fibroblasts^12,13^. Moreover, IL-4 drives the differentiation of naïve CD4^+^ T cells into IL-4-secreting Th2 cells, thus perpetuating the Th2 and pro-fibrotic responses^14^. Cytokines and chemokines that enhance Th2 immune responses also play important roles in SSc. For instance, IL-6, which is overexpressed in SSc patients, contributes to the development of SSc by driving Th2 differentiation as well as promoting collagen production in fibroblasts^15–18^. Taken together, these studies suggest that Th2 dominance is a key immunological feature of SSc that directly and indirectly promotes fibrosis.

IL-31 is a member of IL-6 cytokine family that was originally described as an inducer of dermatitis in mice^19^. IL-31 is mainly produced by Th2 cells and is expressed in a variety of cells, including fibroblasts, keratinocytes, and macrophages^20,21^. Intracellular transmission of IL-31 signaling is mediated by a heterodimeric receptor consisting of IL-31 receptor A (IL-31RA) and oncostatin M receptor (OSMR). IL-31RA is unique to the IL-31 receptor, whereas OSMR is shared by a receptor complex for oncostatin M^19^. Within these two receptor subunits, IL-31 binds predominantly to IL-31RA. Binding of IL-31 to the IL-31 receptor complex activates JAK/STAT, PI3K/AKT, and other signaling pathways^22–24^, leading to a wide range of immune responses.

IL-31 has been closely associated with Th2-dominant diseases. Indeed, previous studies have shown increased expression of IL-31 in Th2-dominant diseases such as allergic asthma, atopic dermatitis, and cutaneous T-cell lymphoma, where IL-31 overexpression is associated with Th2 responses^25–30^. Of note, nemolizumab, functionally blocking monoclonal antibody (mAb) against IL-31RA, has been shown to improve the skin manifestations of atopic dermatitis in a phase II trial, suggesting the potential of IL-31 as a therapeutic target^31^. In addition, recent studies have suggested the association of IL-31 with liver cirrhosis^32^. In the context of SSc, IL-31 expression is increased in fibrotic lungs of BLM-SSc mice^33^. Furthermore, Yaseen et al. have shown that IL-31 and IL-31RA are up-regulated in patients with SSc^34^. They have also demonstrated the pro-fibrotic effects of IL-31 in human dermal fibroblasts (DFs) and SSc model mice. These backgrounds led us to further explore the roles of IL-31 in SSc and its potential as a therapeutic target.

Here, we show that both IL-31 and IL-31RA are overexpressed in DFs from SSc patients and IL-31 promotes the expression of collagen and Th2-inducing cytokines. Moreover, we demonstrate that inhibiting IL-31 signaling by anti-IL-31RA mAb ameliorates fibrosis and Th2 polarization in BLM-SSc mice, providing a rationale for targeting IL-31 in the treatment of SSc.

## Results

### Serum IL-31 levels correlate with fibrosis and Th2 polarization in SSc

**Fig. 1:**
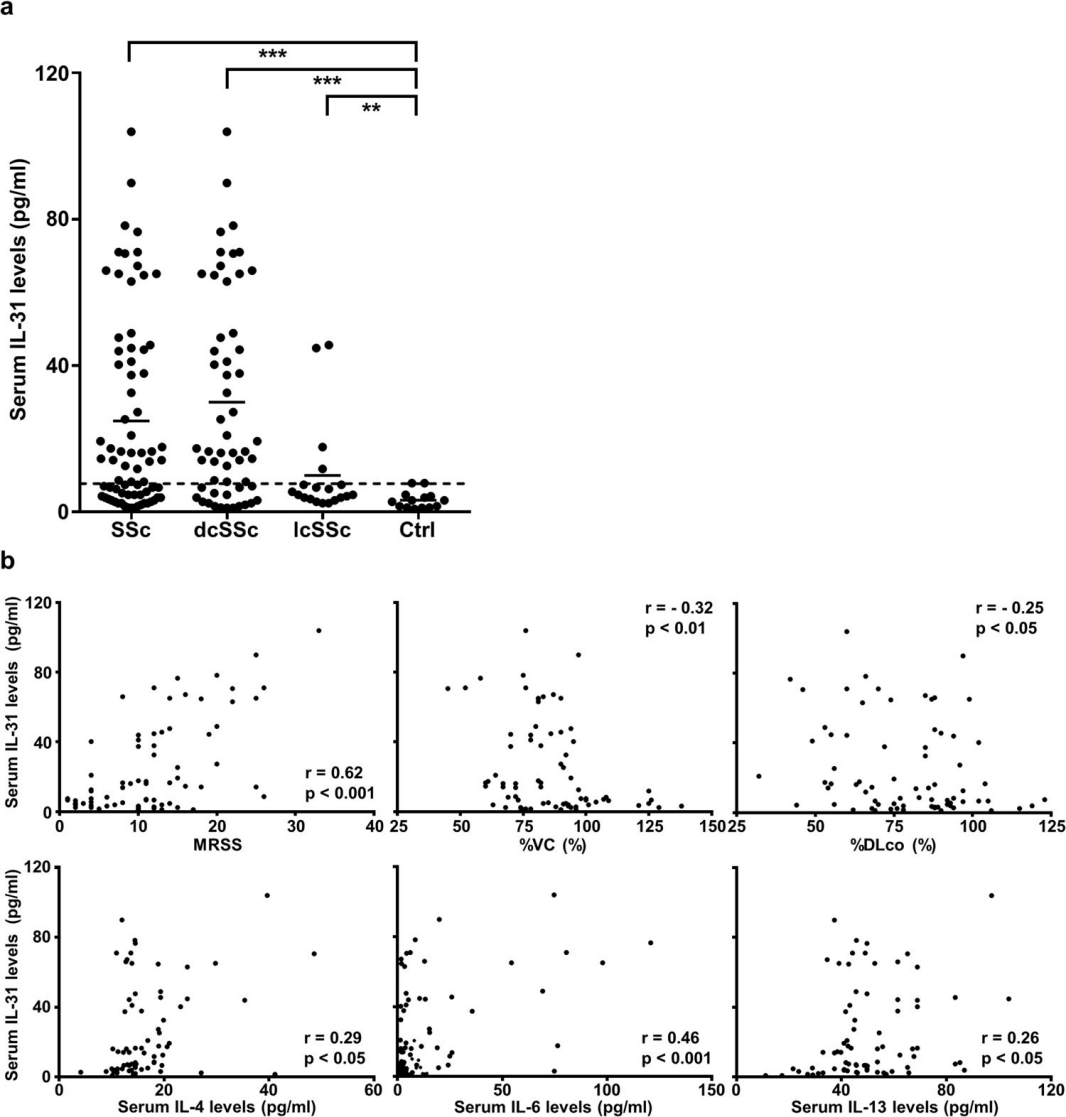
Serum IL-31 levels correlated with fibrosis and Th2 polarization in SSc. **a** Serum IL-31 levels were determined in dcSSc patients (*n* = 55), lcSSc patients (*n* = 19), and healthy controls (*n* = 14) by a specific ELISA kit. The horizontal lines represent the mean values. The broken line represents the cut-off value (mean + 2 SD of the healthy controls). **p < 0.01 and ***p < 0.001 vs. healthy controls. Significance was evaluated by two-tailed Mann–Whitney *U* test. Exact *p* values = 0.00002 (SSc vs. Ctrl), 0.000008 (dcSSc vs. Ctrl), 0.008 (lcSSc vs. Ctrl). Ctrl; healthy controls. **b** Correlations between serum IL-31 levels and the following parameters were analyzed in SSc patients (*n* = 74): MRSS, %VC, %DLco, serum IL-4 levels, serum IL-6 levels, and serum IL-13 levels. Correlations were assessed by Spearman’s rank correlation test. Exact *p* values = 0.000000005 (MRSS); 0.005 (%VC); 0.033 (%DLco); 0.012 (IL-4); 0.00003 (IL-6); 0.026 (IL-13). Source data are provided as a Source Data file.

Initially, we examined IL-31 levels in the sera of 74 SSc patients and 14 healthy controls by enzyme-linked immunosorbent assay (ELISA). Serum IL-31 levels were significantly elevated in SSc patients compared with healthy controls (Fig. 1a). Of note, circulating IL-31 was more abundant in diffuse cutaneous SSc (dcSSc) patients than in limited cutaneous SSc (lcSSc) patients. Next, SSc patients were classified into two groups according to the cut-off value of serum IL-31 levels set at 7.72 pg/ml (mean + 2 SD of healthy controls; Table 1). SSc patients with elevated serum IL-31 levels had higher frequencies of dcSSc, pulmonary fibrosis, and esophagus involvement, all of which are the clinical consequences of fibrosis, when compared to the patients with normal IL-31 levels. Indeed, SSc patients with elevated IL-31 levels were characterized by severe dermal and pulmonary fibrosis, with higher modified Rodnan total skin thickness score^35^ (MRSS) and lower percentage predicted values of vital capacity (%VC) and diffusing capacity for carbon monoxide (%DLco). In addition, SSc patients with elevated IL-31 levels had higher serum levels of IL-4, IL-6, and IL-13, the key cytokines associated with Th2 responses and overexpressed in SSc^3^. Consistently, serum IL-31 levels positively correlated with MRSS, negatively correlated with %VC and %DLco, and positively correlated with serum levels of IL-4, IL-6, and IL-13 (Fig. 1b). Thus, IL-31 overproduction in the sera of SSc patients correlated with fibrosis and Th2 up-regulation, the major features of the disease.

**Table 1:** Clinical and laboratory features of SSc patients.

Characteristic	Elevated IL-31 levels (*n* = 43)	Normal IL-31 levels (*n* = 31)
Sex, no. of women/men	40/3	27/4
Age at onset, median (quartiles) years	44 (30–63)	53 (38–67)
Disease duration, median (quartiles) years	1 (0–4)	1 (0–3)
Disease pattern, No. with dcSSc/lcSSc	39/4***	16/15
MRSS, median (quartiles) points	14 (10–20)***	8 (3–12)
*Cutaneous vascular symptoms*		
Raynaud’s phenomenon	93	90
Nail fold bleeding	67	52
Telangiectasia	53	45
Pitting scars	51	45
Digital ulcers	37	32
*Organ involvement*		
Lungs		
Pulmonary fibrosis	84***	35
%VC, median (quartiles) %	81 (70–90)***	93 (78–104)
%DLco, median (quartiles) %	74 (60–88)**	88 (74–94)
Pulmonary hypertension	5	6
Esophagus	86*	65
Kidneys	5	0
*Laboratory features*		
IL-4, median (quartiles) pg/ml	16.2 (13.4–19.8)**	13.4 (11.0–14.7)
IL-6, median (quartiles) pg/ml	6.13 (2.7–25.0)**	2.50 (1.8–4.3)
IL-13, median (quartiles) pg/ml	49.8 (42.2–65.3)*	45.7 (31.2–53.8)

Unless noted otherwise, values are in percentage. *p < 0.05, **p < 0.01, ***p < 0.001 vs. patient with SSc with normal serum IL-31 levels. Significance was determined by Fisher’s exact probability test for comparison of proportions and two-tailed Mann–Whitney *U* test for quantitative variables. Source data are provided as a Source Data file.

### Both IL-31 and IL-31RA are overexpressed in SSc DFs

**Fig. 2:**
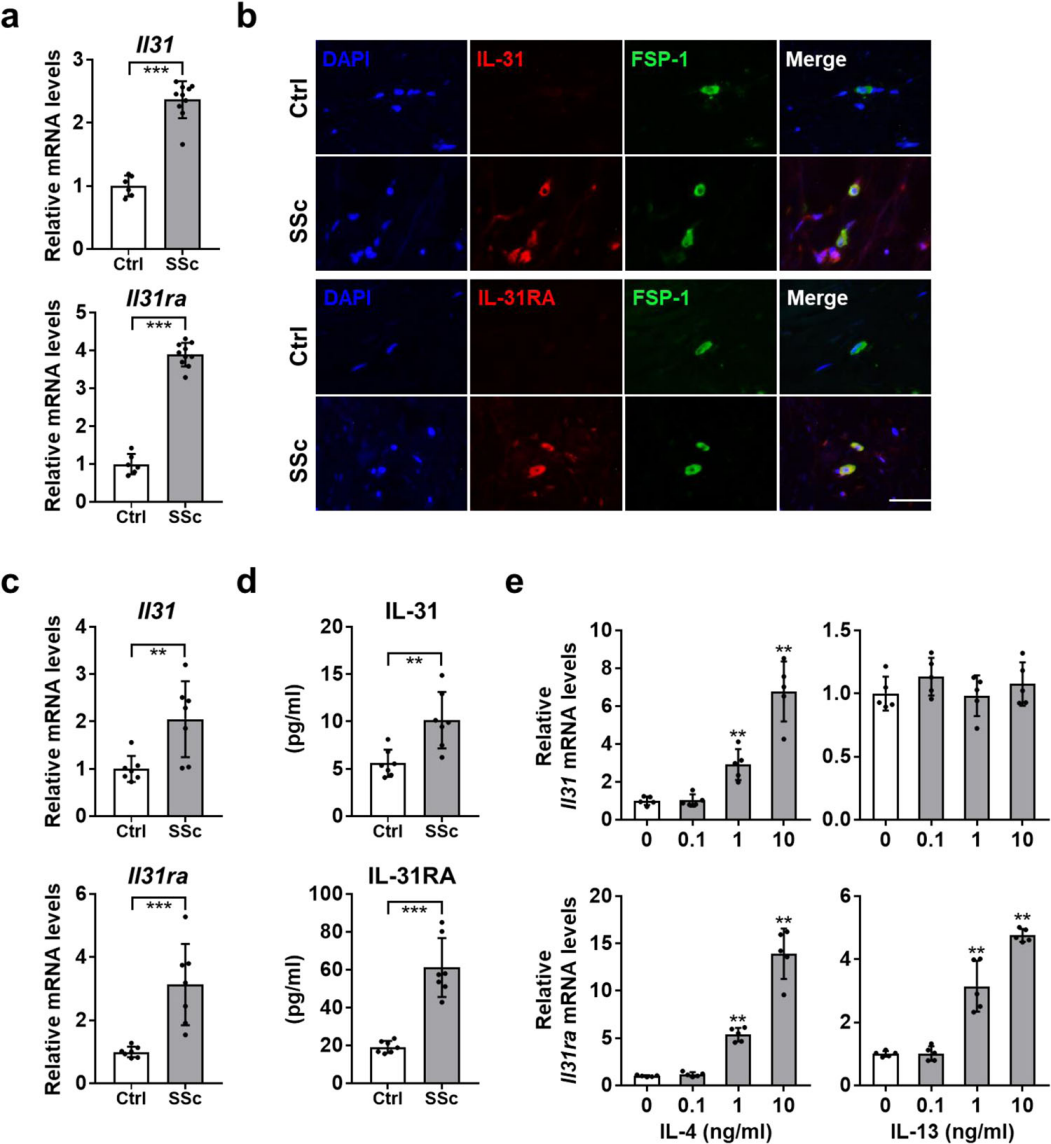
IL-31 and IL-31RA were up-regulated in skin and DFs of SSc patients. **a** Relative mRNA expression levels of *Il31* and *Il31ra* were analyzed by real-time PCR in the skin of SSc patients (*n* = 10) and healthy controls (*n* = 6). Exact p values = 0.0002 (*Il31*); 0.0002 (*Il31ra*). Relative fold differences = 2.37 (*Il31*); 3.89 (*Il31ra*). **b** Representative double-immunofluorescence images for IL-31 (top, red), IL-31RA (bottom, red), FSP-1 (green), and nuclei (DAPI, blue) in the skin of SSc patients and healthy controls (scale bar=20 µm). The results shown are representative of three independent experiments with similar results. **c**, **d**. Relative mRNA levels (**c**) and protein levels (**d**) of IL-31 and IL-31RA in DFs from SSc patients and healthy controls (*n* = 7, respectively) were assessed by real-time PCR and ELISA, respectively. Exact p values = 0.007 (*Il31*); 0.0006 (*Il31ra*); 0.004 (IL-31); 0.0006 (IL-31RA). Relative fold differences = 2.05 (*Il31*); 3.13 (*Il31ra*). e. Relative mRNA expression levels of *Il31* and *Il31ra* were examined by real-time PCR in SSc DFs stimulated with IL-4 or IL-13 (*n* = 5, respectively). Exact p values (IL-4 0.1 ng/ml vs. media, IL-4 1 ng/ml vs. media, IL-4 10 ng/ml vs. media, IL-13 0.1 ng/ml vs. media, IL-13 1 ng/ml vs. media, IL-13 10 ng/ml vs. media) = 0.841, 0.008, 0.008, 0.151, 0.841, 0.548 (*Il31*); 0.310, 0.008, 0.008, 0.999, 0.008, 0.008 (*Il31ra*). Relative fold differences (IL-4 0.1 ng/ml, IL-4 1 ng/ml, IL-4 10 ng/ml, IL-13 0.1 ng/ml, IL-13 1 ng/ml, IL-13 10 ng/ml) = 1.02, 2.92, 6.78, 1.13, 0.98, 1.08 (*Il31*); 1.16, 5.36, 13.91, 1.01, 3.15, 4.76 (*Il31ra*). Data are presented as mean ± SD. *p < 0.05, **p < 0.01, and ***p < 0.001 vs. healthy controls (**a**, **c**, and **d**) or media (**e**). Significance was determined by two-tailed Mann-Whitney *U* test. Ctrl, healthy controls. Source data are provided as a Source Data file.

Next, we assessed mRNA levels of *Il31* and *Il31ra* in the skin of 10 SSc patients and six healthy controls by real-time polymerase chain reaction (PCR). The SSc lesional skin showed significantly higher mRNA levels of *Il31* and *Il31ra* compared with the skin of healthy controls (Fig. 2a). We next performed double-immunofluorescence staining to evaluate the expression and localization of IL-31 and IL-31RA protein in the SSc lesional skin (Fig. 2b). IL-31 and IL-31RA were colocalized with fibroblast-specific protein 1 (FSP-1), a fibroblast marker, in SSc lesional skin, indicating the expression of IL-31 and IL-31RA in SSc DFs. We did not detect colocalization of IL-31 or IL-31RA with FSP-1 in the skin of healthy controls. In SSc lesional skin, IL-31 was also colocalized with CD4 (Supplementary Fig. 1a), which is consistent with the previous studies showing IL-31 expression in Th2 cells^19,20^. Within the SSc subgroups, IL-31^+^CD4^+^ T cells were more abundant in the skin of dcSSc patients than in the skin of lcSSc patients (Supplementary Fig. 1b). In cultured SSc DFs, IL-31 and IL-31RA expression was significantly elevated at both mRNA and protein levels compared with DFs from healthy controls (Fig. 2c, d). Moreover, *Il31* expression was enhanced by IL-4, and *Il31ra* expression was enhanced by IL-4 and IL-13 in SSc DFs (Fig. 2e), implying that Th2 dominance might contribute to the overexpression of IL-31 and IL-31RA in SSc DFs. We also detected the increased secretion of IL-31 from SSc DFs that were stimulated with IL-4 (Supplementary Fig. 2a). Collectively, both IL-31 and IL-31RA expression was up-regulated in SSc DFs, suggesting a role of the IL-31/IL-31RA axis in SSc fibrosis.

### IL-31 promotes collagen and pro-Th2 cytokine production in SSc DFs

**Fig. 3:**
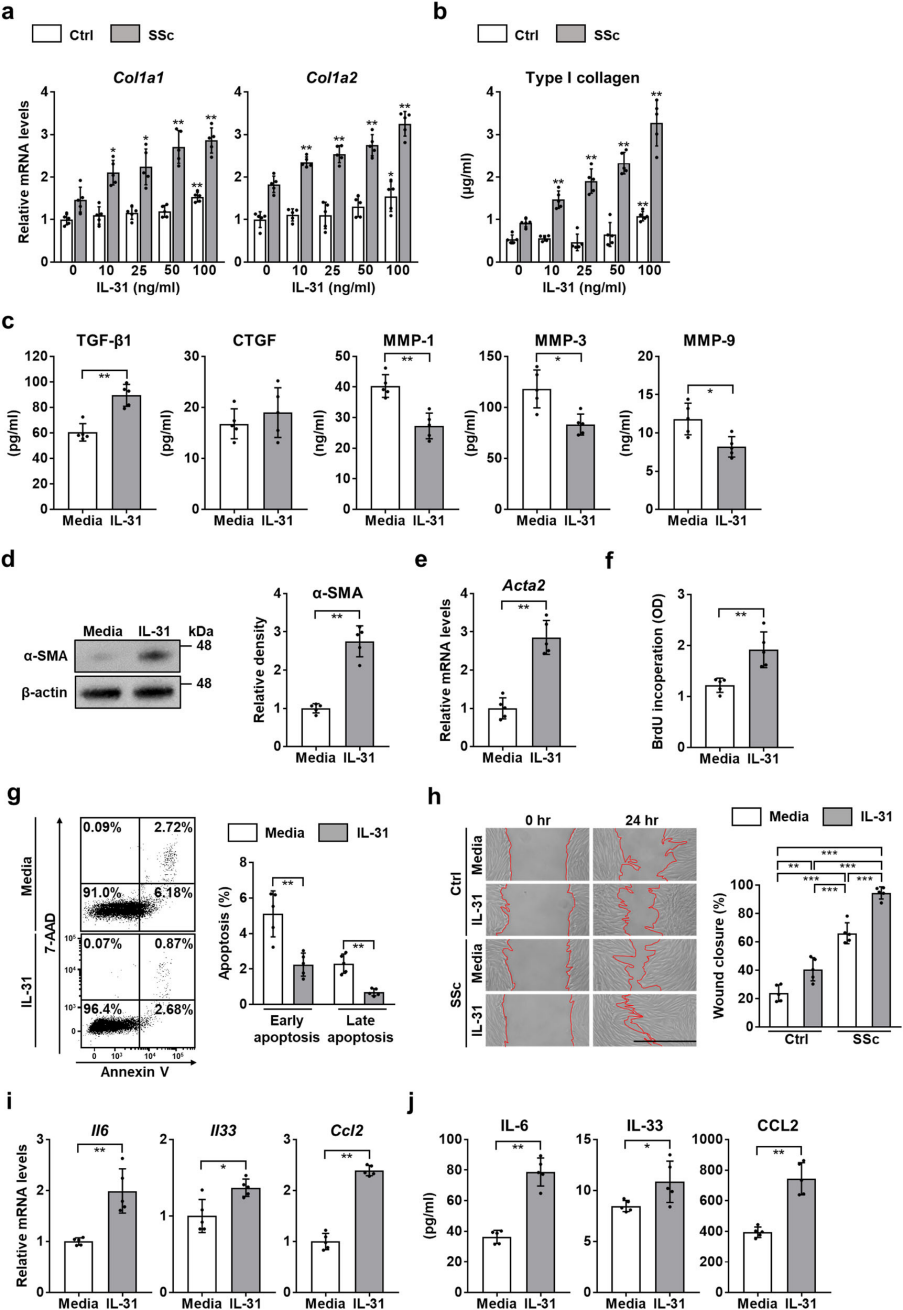
IL-31 induced SSc-like phenotype in DFs. **a**, **b** DFs from dcSSc patients and healthy controls were cultured for 24 h with rhIL-31 (0, 10, 25, 50, or 100 ng/ml) to examine *Col1a1* and *Col1a2* mRNA expression (**a**) and type I collagen production (**b**) by real-time PCR and ELISA, respectively. Exact *p* values (Ctrl + IL-31 10 ng/ml vs. Ctrl, Ctrl + IL-31 25 ng/ml vs. Ctrl, Ctrl + IL-31 50 ng/ml vs. Ctrl, Ctrl + IL-31 100 ng/ml vs. Ctrl, SSc + IL-31 10 ng/ml vs. SSc, SSc + IL-31 25 ng/ml vs. SSc, SSc + IL-31 50 ng/ml vs. SSc, SSc + IL-31 100 ng/ml vs. SSc) = 0.690, 0.095, 0.056, 0.008, 0.032, 0.016, 0.008, 0.008 (*Col1a1*); 0.421, 0.421, 0.222, 0.016, 0.008, 0.008, 0.008, 0.008 (*Col1a2*); 0.508, 0.127, 0.841, 0.008, 0.008, 0.008, 0.008, 0.008 (Type I collagen). Relative fold differences (Ctrl + IL-31 10 ng/ml, Ctrl + IL-31 25 ng/ml, Ctrl + IL-31 50 ng/ml, Ctrl + IL-31 100 ng/ml, SSc + IL-31 10 ng/ml, SSc + IL-31 25 ng/ml, SSc + IL-31 50 ng/ml, SSc + IL-31 100 ng/ml) = 1.10, 1.16, 1.19, 1.53, 1.47, 2.10, 2.24, 2.71, 2.86 (*Col1a1*); 1.11, 1.10, 1.31, 1.54, 1.83, 2.35, 2.54, 2.75, 3.25 (*Col1a2*). **c** Protein levels of TGF-β1, CTGF, MMP-1, MMP-3, and MMP-9 were assessed by ELISA in the supernatants of dcSSc DFs treated with or without rhIL-31 (50 ng/ml). Exact p values = 0.008 (TGF-β1), 0.587 (CTGF), 0.008 (MMP-1), 0.016 (MMP-3), 0.024 (MMP-9). **d** Representative western blot analysis of α-SMA in dcSSc DFs treated with or without rhIL-31 (50 ng/ml; left). Quantification of protein expression normalized to β-actin (right). Exact *p* value = 0.008. **e** Relative mRNA expression level of *Acta2* was evaluated by real-time PCR in dcSSc DFs treated with or without rhIL-31 (50 ng/ml). Exact *p* value = 0.008. Relative fold difference = 2.85. **f** BrdU incorporation was quantified by ELISA in dcSSc DFs treated with or without rhIL-31 (50 ng/ml). The absorbance at 450 nm was measured. Exact *p* value = 0.008. **g** DFs from dcSSc patients were treated with or without rhIL-31 (50 ng/ml) and analyzed for apoptosis by flow cytometry. Representative dot plots were shown (left). Annexin-V^+^, 7-AAD^−^ cells were considered early apoptotic cells, and Annexin-V^+^, 7-AAD^+^ cells were considered late apoptotic cells, respectively (right). Exact p values (IL-31 vs. media) = 0.008 (early apoptosis); 0.008 (late apoptosis). **h** DFs from dcSSc patients and healthy controls were pretreated with mitomycin C, scratched to make a wound, and incubated for 24 h with rhIL-31 (50 ng/ml) or media alone. Representative microscopic images were shown (left, scale bar=500 µm). Red lines show the borders of the wounds. Wound closure was expressed as the percentage of wound reduction from the original wound (right). Exact p values (Ctrl + media vs. Ctrl + IL-31, Ctrl + media vs. SSc + media, Ctrl + media vs. SSc + IL-31, Ctrl + IL-31 vs. SSc + media, Ctrl + IL-31 vs. SSc + IL-31, SSc + media vs. SSc + IL-31) = 0.005, 0.0000001, 0.00000000006, 0.00008, 0.000000004, 0.00002. **i**, **j** DFs obtained from dcSSc patients were cultured with rhIL-31 (50 ng/ml) or media alone for 24 h, and expression levels of IL-6, IL-33, and CCL2 were measured by real-time PCR (**i**) and ELISA (**j**). Exact p values = 0.008 (*Il6*); 0.032 (*Il33*); 0.008 (*Ccl2*); 0.008 (IL-6); 0.032 (IL-33); 0.008 (CCL2). Relative fold differences = 1.99 (*Il6*); 1.37 (*Il33*); 2.39 (*Ccl2*). n = 5. Data are presented as mean ± SD. *p < 0.05, **p < 0.01, and ***p < 0.001 vs. unstimulated Ctrl or SSc fibroblasts (**a**, **b**) or media (**c–g**, **i**, and **j**). Significance was determined by two-tailed Mann-Whitney *U* test (**a–g**, **i**, and **j**) and one-way analysis of variance followed by Tukey’s post hoc comparison test (h). Ctrl, healthy controls; OD, optical density. Source data are provided as a Source Data file.

To investigate the role of IL-31 in SSc DFs, we subsequently stimulated DFs from SSc patients and healthy controls with recombinant human (rh) IL-31. In SSc DFs, rhIL-31 significantly and dose-dependently increased the mRNA levels of *Col1a1* and *Col1a2* (Fig. 3a), which encode type I collagen, the major extracellular matrix component of skin^36^. By contrast, in healthy control DFs, the effect of rhIL-31 on collagen expression was modest, with *Col1a1* and *Col1a2* levels increased only when stimulated with a high concentration of rhIL-31 (100 ng/ml). Similarly, rhIL-31 enhanced type I collagen production from SSc DFs in a dose-dependent manner, whereas in healthy control DFs, type I collagen production was induced only at a high concentration of rhIL-31 (100 ng/ml; Fig. 3b). These results suggest that IL-31 is a direct inducer of collagen synthesis in SSc DFs, where IL-31RA is highly expressed. We also observed that the conditioned media of SSc DFs significantly increased the production of type I collagen in SSc DFs via IL-31 signaling (Supplementary Fig. 2b, c). Furthermore, rhIL-31 increased the expression of transforming growth factor (TGF)-β1 and decreased the expression of MMP-1, MMP-3, and MMP-9 in SSc DFs (Fig. 3c). Because TGF-β1 enhances collagen production in fibroblasts while MMPs promote its degradation^37,38^, IL-31 might contribute to the cytokine environment that facilitates the excessive collagen deposition in SSc DFs. Stimulation with rhIL-31 also elevated the expression of α-smooth muscle actin (α-SMA) at both mRNA and protein levels (Fig. 3d, e), indicating that IL-31 promotes the transdifferentiation of SSc DFs to myofibroblasts. Moreover, rhIL-31 promoted proliferation and migration, and suppressed apoptosis of SSc DFs (Fig. 3f–h), thus enhancing the characteristic features of SSc DFs that contribute to the excessive fibrosis in SSc^39^. Overall, IL-31 enhanced SSc-like phenotype as well as promoted collagen production in SSc DFs.

Apart from the excessive collagen synthesis, SSc DFs are associated with distinct profiles of cytokines and chemokines that contribute to the immune abnormalities of the disease^40–42^. Therefore, to explore the effect of IL-31 on cytokine and chemokine expression in SSc DFs, we stimulated SSc DFs with rhIL-31 and evaluated the expression of the following cytokines that are expressed in DFs and drive Th2 immune responses: IL-6, IL-33, and CC chemokine ligand (CCL) 2^40–42^. As shown in Fig. 3i, j, rhIL-31 significantly increased the expression of IL-6, IL-33, and CCL2 at both mRNA and protein levels in SSc DFs. Thus, IL-31 also promoted pro-Th2 cytokine expression in SSc DFs.

**Fig. 4:**
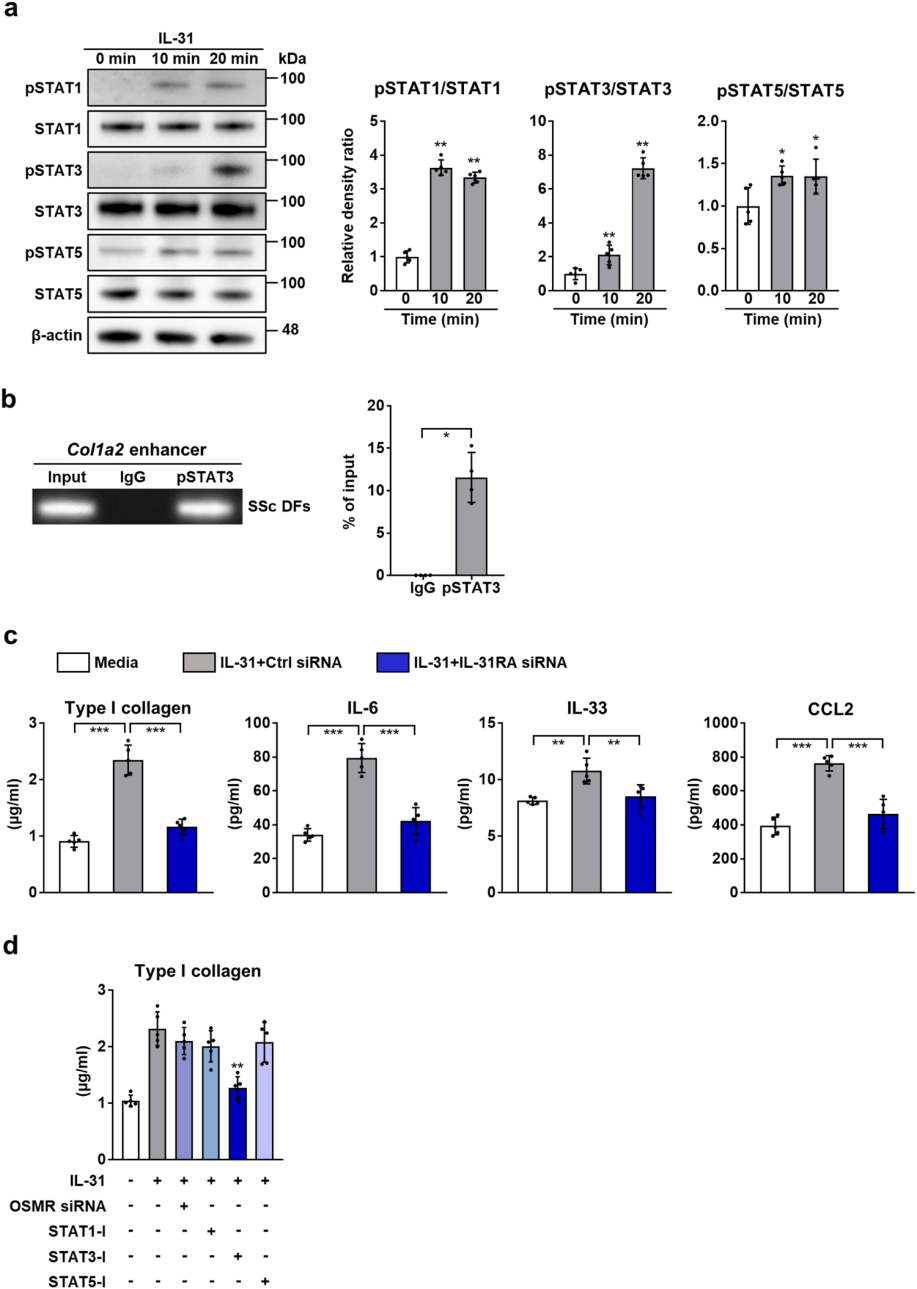
IL-31 exerted its effects through IL-31RA and STAT3. **a** Representative western blot analysis of STAT1, STAT3, STAT5 and their phosphorylated forms in dcSSc DFs treated with rhIL-31 (50 ng/ml; left). Quantification of protein expression normalized to β-actin (right). Exact p values (10 min vs. 0 min, 20 min vs. 0 min) = 0.008, 0.008 (pSTAT1/STAT1); 0.008, 0.008 (pSTAT3/STAT3); 0.016, 0.040 (pSTAT5/STAT5). Relative fold differences (10 min, 20 min) = 3.63, 3.34 (pSTAT1/STAT1); 2.11, 7.23 (pSTAT3/STAT3); 1.36, 1.35 (pSTAT5/STAT5). **b** Representative ChIP assay on dcSSc DFs assessing the binding of phosphorylated STAT3 to the *Col1a2* enhancer (left). Immunoprecipitated chromatin was analyzed by real-time PCR using specific primers for the HS4 region of the *Col1a2* enhancer (right). Exact *p* value = 0.029. *n* = 4 biologically independent experiments. **c** Protein levels of type I collagen, IL-6, IL-33, and CCL2 were evaluated by ELISA in the supernatants of dcSSc DFs that were transfected with IL-31RA siRNA and treated with rhIL-31 (50 ng/ml). Exact p values (media vs. IL-31 + Ctrl siRNA, media vs. IL-31 + IL-31RA siRNA, IL-31 + Ctrl siRNA vs. IL-31 + IL-31RA siRNA) = 0.00000009, 0.111, 0.0000008 (Type I collagen); 0.0000009, 0.202, 0.000007 (IL-6); 0.002, 0.766, 0.005 (IL-33); 0.000003, 0.222, 0.00002 (CCL2). **d** Protein levels of Type I collagen were measured by ELISA in the supernatants of dcSSc DFs that were transfected with OSMR siRNA or treated with inhibitors for STAT1 (STAT1-I: Fludarabine; 50 µM), STAT3 (STAT-I: Stattic; 5 µM), or STAT5 (STAT5-I: CAS 285986-31-4; 50 µM), followed by the treatment with rhIL-31 (50 ng/ml). Exact *p* values = 0.238 (IL-31 + OSMR siRNA vs. IL-31), 0.175 (IL-31 + STAT1-I vs. IL-31), 0.008 (IL-31 + STAT3-I vs. IL-31), 0.548 (IL-31 + STAT5-I vs. IL-31). *n* = 5 biologically independent experiments, unless otherwise noted. Data are presented as mean ± SD. *p < 0.05, **p < 0.01, and ***p < 0.001. Significance was determined using two-tailed Mann-Whitney *U* test (**a**, **b**, and d) and one-way analysis of variance followed by Tukey’s post hoc comparison test (**c**). pSTAT1, phosphorylated STAT1; pSTAT3, phosphorylated STAT3; pSTAT5, phosphorylated STAT5. Source data are provided as a Source Data file.

Subsequently, we explored the signaling pathways through which IL-31 exerts its effects on SSc DFs. Western blot analysis revealed that rhIL-31 induced the phosphorylation of STAT3 in SSc DFs (Fig. 4a), which is in accordance with the study by Yaseen et al. ^34^. Stimulation with rhIL-31 also induced the phosphorylation of STAT1 and STAT5 in SSc DFs, but to a lesser degree than STAT3. Because a previous study has demonstrated that the STAT3 binds to the enhancer region of *Col1a2* and is important for the excessive collagen production in SSc fibroblasts^43^, we hypothesized that a pathway involving IL-31, STAT3, and *Col1a2* would promote collagen production in SSc DFs. To examine this hypothesis, we performed chromatin immunoprecipitation (ChIP) assay. STAT3 binding site was set at the HS4 region as previously reported^43^. As demonstrated in Fig. 4b, the binding of STAT3 to the HS4 legion of the *Col1a2* enhancer was observed in SSc DFs.

Furthermore, we explored whether the effects of IL-31 on SSc DFs are mediated by the signaling through IL-31RA. Knock-down of IL-31RA with small interfering RNA (siRNA) significantly decreased the expression of type I collagen, IL-6, IL-33, and CCL2 that was induced by IL-31(Fig. 4c). Because the mRNA expression of *OSMR*, the other receptor subunit comprising the IL-31 receptor complex, was elevated in SSc (Supplementary Fig. 3), we also investigated whether and to what extent the signaling through OSMR contributed to the increased collagen expression induced by IL-31 in SSc DFs. As shown in Fig. 4d, knock-down of OSMR did not significantly change the expression of type I collagen in SSc DFs that were stimulated with rhIL-31. Therefore, signaling through IL-31RA seems to be more important for the induction of type I collagen by IL-31 in SSc DFs than signaling through OSMR. We also observed that a selective STAT3 inhibitor significantly decreased the collagen production from SSc DFs induced by rhIL-31 (Fig. 4d). Selective inhibitors of STAT1 or STAT5 did not significantly affect the collagen production. Therefore, the signaling through STAT3 indeed seems to play an important role in the induction of collagen by IL-31. Overall, these results suggest that the effects of IL-31 on SSc DFs are mainly mediated by IL-31RA and STAT3.

### IL-31 promotes skin and lung fibrosis in BLM-SSc mice

**Fig. 5:**
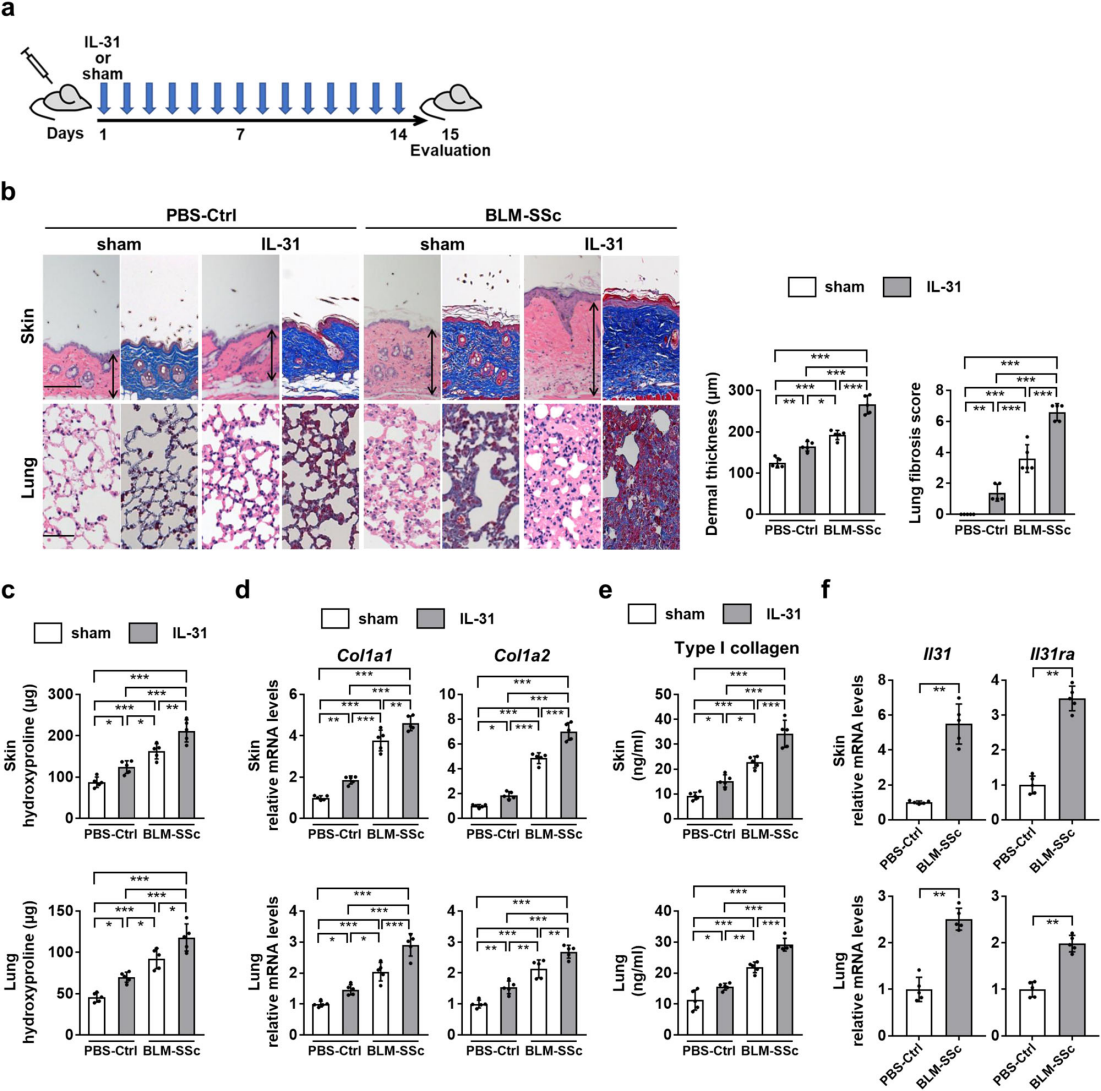
IL-31 augmented skin and lung fibrosis in BLM-SSc mice. **a** BLM-SSc and PBS-treated control (PBS-Ctrl) mice were administered with either rmIL-31 or saline as a sham daily from day 1 to 14, and were analyzed on day 15. **b** Representative histological sections of the skin and lungs stained with hematoxylin and eosin (left) and Masson trichrome (right) were shown (horizontal scale bars=100 μm in skin; 20 µm in lung). Vertical bars with arrows represent dermal thickness. Dermal thickness and lung fibrosis score were assessed histologically. Exact p values (PBS-Ctrl + sham vs. PBS-Ctrl + IL-31, PBS-Ctrl + sham vs. BLM-SSc + sham, PBS-Ctrl + sham vs. BLM-SSc + IL-31, PBS-Ctrl + IL-31 vs. BLM-SSc + sham, PBS-Ctrl + IL-31 vs. BLM-SSc + IL-31, BLM-SSc + sham vs. BLM-SSc + IL-31) = 0.003, 0.00001, 0.0000000005, 0.049, 0.00000006, 0.000004 (dermal thickness); 0.009, 0.0000003, 0.00000000004, 0.0001, 0.000000001, 0.000003 (lung fibrosis score). **c** Hydroxyproline contents of skin and lung samples. Exact p values (PBS-Ctrl + sham vs. PBS-Ctrl + IL-31, PBS-Ctrl + sham vs. BLM-SSc + sham, PBS-Ctrl + sham vs. BLM-SSc + IL-31, PBS-Ctrl + IL-31 vs. BLM-SSc + sham, PBS-Ctrl + IL-31 vs. BLM-SSc + IL-31, BLM-SSc + sham vs. BLM-SSc + IL-31) = 0.036, 0.00007, 0.0000001, 0.027, 0.00001, 0.004 (skin); 0.015, 0.00003, 0.0000001, 0.025, 0.00002, 0.011 (lung). **d** Relative mRNA expression levels of *Col1a1* and *Col1a2* in the skin and lungs were evaluated by real-time PCR. Exact *p* values (PBS-Ctrl + sham vs. PBS-Ctrl + IL-31, PBS-Ctrl + sham vs. BLM-SSc + sham, PBS-Ctrl + sham vs. BLM-SSc + IL-31, PBS-Ctrl + IL-31 vs. BLM-SSc + sham, PBS-Ctrl + IL-31 vs. BLM-SSc + IL-31, BLM-SSc + sham vs. BLM-SSc + IL-31) = 0.004, 0.000000002, 0.00000000004, 0.0000004, 0.000000003, 0.005 (*Col1a1*, skin); 0.033, 0.000000002, 0.000000000002, 0.00000006, 0.00000000002, 0.000005 (*Col1a2*, skin); 0.046, 0.00004, 0.00000001, 0.011, 0.0000006, 0.0003 (*Col1a1*, lung); 0.006, 0.000002, 0.000000007, 0.002, 0.000001, 0.004 (*Col1a2*, lung). Relative fold differences (PBS-Ctrl + IL-31, BLM-SSc + sham, BLM-SSc + IL-31) = 1.86, 3.78, 4.61 (*Col1a1*, skin); 1.86, 4.87, 7.03 (*Col1a2*, skin); 1.47, 2.05, 2.91 (*Col1a1*, lung); 1.54, 2.14, 2.69 (*Col1a2*, lung). **e** Protein levels of type I collagen in the skin and lungs were assessed by ELISA. Exact p values (PBS-Ctrl + sham vs. PBS-Ctrl + IL-31, PBS-Ctrl + sham vs. BLM-SSc + sham, PBS-Ctrl + sham vs. BLM-SSc + IL-31, PBS-Ctrl + IL-31 vs. BLM-SSc + sham, PBS-Ctrl + IL-31 vs. BLM-SSc + IL-31, BLM-SSc + sham vs. BLM-SSc + IL-31) = 0.048, 0.00004, 0.00000001, 0.011, 0.0000006, 0.0003 (skin); 0.029, 0.000005, 0.000000004, 0.002, 0.0000002, 0.0004 (lung). **f** Relative mRNA expression levels of *Il31* and *Il31ra* in the skin and lungs were evaluated by real-time PCR. Exact p values = 0.008 (*Il31*, skin); 0.008 (*Il31ra*, skin); 0.008 (*Il31*, lung); 0.008 (*Il31ra*, lung). Relative fold differences = 5.49 (*Il31*, skin); 3.48 (*Il31ra*, skin); 2.51 (*Il31*, lung); 1.98 (*Il31ra*, lung). *n* = 5. Data are shown as mean ± SD. *p < 0.05, **p < 0.01, and ***p < 0.001. Significance was determined using one-way analysis of variance followed by Tukey’s post hoc comparison test (**b**–**e**) and two-tailed Mann-Whitney *U* test (f). The results shown are representative of three independent experiments with similar results. PBS-Ctrl, PBS-treated control. Source data are provided as a Source Data file.

To investigate the effect of IL-31 on fibrosis in vivo, we subcutaneously injected BLM-SSc mice and PBS-treated control mice with recombinant mouse IL-31 (rmIL-31; Fig. 5a). Administration of rmIL-31 significantly exacerbated skin and lung fibrosis of BLM-SSc mice (Fig. 5b, c). Consistently, BLM-SSc mice treated with rmIL-31 showed significantly increased expression of *Col1a1*, *Col1a2*, and type I collagen, compared with BLM-SSc mice treated with sham (Fig. 5d, e). In PBS-treated control mice, rmIL-31 induced skin and lung fibrosis significantly but to a lesser extent compared with BLM-SSc mice. We also observed the up-regulation of both IL-31 and IL-31RA in the fibrotic skin and lungs of BLM-SSc mice compared with PBS-treated control mice (Fig. 5f and Supplementary Fig. 4). Therefore, potent induction of the skin and lung fibrosis by rmIL-31 in BLM-SSc mice may be attributed to the abundant expression of IL-31RA in these tissues. Next, we explored the cell types expressing IL-31 in lung tissue (Supplementary Fig. 5a). In BLM-SSc mice, *Il31* was primarily expressed by fibroblasts, where the knock-down of IL-31 with siRNA decreased the release of type I collagen (Supplementary Fig. 5b). Therefore, the autocrine expression of *Il31*in fibroblasts might play an important role for the excessive collagen production in the lungs of BLM-SSc mice. Similar results were obtained with *Il31ra*, which was also mainly expressed by fibroblasts in the lungs of BLM-SSc mice and whose knock-down in fibroblasts decreased the collagen release (Supplementary Fig. 5c, d). These results indicate the functional relevance of IL-31 and IL-31RA expression by fibroblasts in the context of BLM-induced lung fibrosis.

**Fig. 6:**
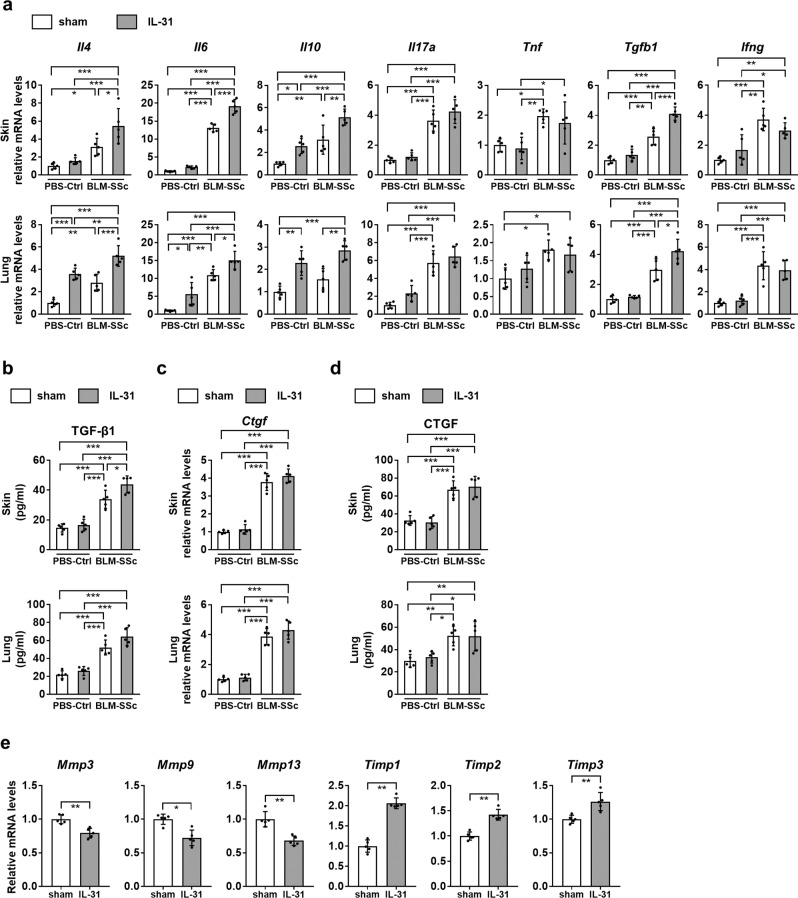
IL-31 up-regulated Th2- and fibrosis-related cytokines in BLM-SSc mice. **a** Relative mRNA expression levels of *Il4*, *Il6*, *Il10*, *Il17a*, *Tnf*, *Tgfb1*, and *Ifng* in the skin and lungs were evaluated by real-time PCR. Exact p values (PBS-Ctrl + sham vs. PBS-Ctrl + IL-31, PBS-Ctrl + sham vs. BLM-SSc + sham, PBS-Ctrl + sham vs. BLM-SSc + IL-31, PBS-Ctrl + IL-31 vs. BLM-SSc + sham, PBS-Ctrl + IL-31 vs. BLM-SSc + IL-31, BLM-SSc + sham vs. BLM-SSc + IL-31) = 0.866, 0.039, 0.00006, 0.162, 0.0003, 0.021 (*Il4*, skin); 0.403, 0.00000000006, 0.0000000000002, 0.0000000003, 0.0000000000004, 0.000001 (*Il6*, skin); 0.034, 0.004, 0.000003, 0.699, 0.0007, 0.007 (*Il10*, skin); 0.926, 0.000007, 0.0000005, 0.00002, 0.000001, 0.344 (*Il17a*, skin); 0.977, 0.012, 0.063, 0.005, 0.029, 0.832 (*Tnf*, skin); 0.572, 0.0001, 0.00000001, 0.001, 0.00000008, 0.0001 (*Tgfb1*, skin); 0.428, 0.00007, 0.002, 0.001, 0.040, 0.361 (*Ifng*, skin); 0.00008, 0.003, 0.0000002, 0.286, 0.008, 0.0002 (*Il4*, lung); 0.023, 0.00001, 0.0000001, 0.007, 0.00002, 0.038 (*Il6*, lung); 0.002, 0.227, 0.00003, 0.085, 0.231, 0.002 (*Il10*, lung); 0.192, 0.000008, 0.000001, 0.0004, 0.00005, 0.686 (*Il17a*, lung); 0.641, 0.013, 0.041, 0.127, 0.321, 0.937 (*Tnf*, lung); 0.967, 0.0002, 0.0000005, 0.0005, 0.000001, 0.014 (*Tgfb1*, lung); 0.973, 0.00003, 0.0001, 0.00007, 0.0003, 0.868 (*Ifng*, lung). Relative fold differences (PBS-Ctrl + IL-31, BLM-SSc + sham, BLM-SSc + IL-31) = 1.55, 3.12, 5.45 (*Il4*, skin); 2.14, 13.09, 19.11 (*Il6*, skin); 2.59, 3.15, 5.16 (*Il10*, skin); 1.22, 3.63, 4.24 (*Il17a*, skin); 0.89, 1.97, 1.74 (*Tnf*, skin); 1.34, 2.57, 4.12 (*Tgfb1*, skin); 1.68, 3.71, 2.98 (*Ifng*, skin); 3.59, 2.81, 5.20 (*Il4*, lung); 5.56, 10.92, 15.12 (*Il6*, lung); 2.29, 1.56, 2.85 (*Il10*, lung); 2.35, 5.74, 6.45 (*Il17a*, lung); 1.27, 1.81, 1.68 (*Tnf*, lung); 1.16, 2.98, 4.22 (*Tgfb1*, lung); 1.22, 4.34, 3.95 (*Ifng*, lung). **b** Protein levels of TGF-β1 were evaluated by ELISA. Exact p values (PBS-Ctrl + sham vs. PBS-Ctrl + IL-31, PBS-Ctrl + sham vs. BLM-SSc + sham, PBS-Ctrl + sham vs. BLM-SSc + IL-31, PBS-Ctrl + IL-31 vs. BLM-SSc + sham, PBS-Ctrl + IL-31 vs. BLM-SSc + IL-31, BLM-SSc + sham vs. BLM-SSc + IL-31) = 0.924, 0.00006, 0.0000003, 0.0002, 0.0000007, 0.022 (skin); 0.805, 0.00003, 0.0000004, 0.0002, 0.000002, 0.076 (lung). **c**, **d** Relative mRNA levels and protein levels of *Ctgf* were evaluated by real-time PCR (**c**) and ELISA (**d**), respectively. Exact p values (PBS-Ctrl + sham vs. PBS-Ctrl + IL-31, PBS-Ctrl + sham vs. BLM-SSc + sham, PBS-Ctrl + sham vs. BLM-SSc + IL-31, PBS-Ctrl + IL-31 vs. BLM-SSc + sham, PBS-Ctrl + IL-31 vs. BLM-SSc + IL-31, BLM-SSc + sham vs. BLM-SSc + IL-31) = 0.912, 0.000000003, 0.0000000005, 0.000000007, 0.000000001, 0.391 (*Ctgf*, skin); 0.967, 0.00000007, 0.000000009, 0.0000001, 0.00000002, 0.393 (*Ctgf*, lung); 0.972, 0.00006, 0.00002, 0.00003, 0.00001, 0.941 (CTGF, skin); 0.933, 0.005, 0.006, 0.017, 0.019, 0.999 (CTGF, lung). Relative fold difference (PBS-Ctrl + IL-31, BLM-SSc + sham, BLM-SSc + IL-31) = 1.14, 3.79, 4.13 (skin); 1.12, 3.86, 4.30 (lung). **e** Relative mRNA expression levels of *Mmp3*, *Mmp9*, *Mmp13*, *Timp1*, *Timp2*, and *Timp3* were assessed in the lungs of BLM-SSc mice by real-time PCR. Exact p values = 0.008 (*Mmp3*); 0.016 (*Mmp9*); 0.008 (*Mmp13*); 0.008 (*Timp1*); 0.008 (*Timp2*); 0.008 (*Timp3*). Relative fold differences = 0.79 (*Mmp3*); 0.72 (*Mmp9*); 0.68 (*Mmp13*); 2.06 (*Timp1*); 1.42 (*Timp2*); 1.26 (*Timp3*). *n* = 5. Data are shown as mean ± SD. *p < 0.05, **p < 0.01, and ***p < 0.001. Significance was determined using one-way analysis of variance followed by Tukey’s post hoc comparison test (a-d) and two-tailed Mann–Whitney *U* test (**e**). The results shown are representative of three independent experiments with similar results. PBS-Ctrl, PBS-treated control. Source data are provided as a Source Data file.

Subsequently, the effect of rmIL-31 on cytokine production in the skin and lungs were examined by real-time PCR and ELISA. The expression of IL-4, IL-6, IL-10, IL-17A, tumor necrosis factor (TNF), TGF-β1, and interferon (IFN)-γ was significantly up-regulated in the skin and lungs of BLM-SSc mice compared with PBS-treated control mice (Fig. 6a, b, Supplementary Fig. 6), as previously reported^10^. Administration of rmIL-31 further increased the expression levels of IL-4, IL-6, IL-10, and TGF-β1 in the skin and lungs of BLM-SSc mice, promoting the overexpression of Th2 and pro-fibrotic cytokines in these tissues. In contrast, the expression levels of the Th1 cytokine IFN-γ, the Th17 cytokine IL-17A, and TNF in BLM-SSc mice were not altered by rmIL-31. Therefore, IL-31 augmented BLM-induced fibrosis with increased expression of Th2 and pro-fibrotic cytokines in the sclerotic skin and lungs. We did not observe the significant change of CTGF expression in skin or lung by rmIL-31 (Fig. 6c, d), which is consistent with the results obtained in SSc DFs (Fig. 3c). In addition, rmIL-31 significantly decreased the mRNA levels of *Mmp3*, *Mmp9*, and *Mmp13* in the lungs of BLM-SSc mice, while it significantly increased the mRNA levels of *Timp1*, *Timp2*, and *Timp3* (Fig. 6e). Because decreased expression of MMPs and increased expression of TIMPs have been associated with the impaired degradation of collagen in SSc^38^, IL-31 further enhanced the altered expression of MMPs and TIMPs in the lungs of BLM-SSc mice that might contribute to the excessive collagen deposition. Taken together, IL-31 augmented BLM-induced fibrosis with increased expression of cytokines that are associated with fibrosis and Th2 responses.

To further explore whether and to what extent the pro-fibrotic effects of IL-31 in vivo are mediated by Th2 responses, we administered an anti-IL-4 receptor α (IL-4 RA) mAb to mice alongside with rmIL-31 and BLM. As shown in Supplementary Fig. 7a, b, anti-IL-4RA mAb significantly attenuated skin and lung fibrosis induced by rmIL-31. However, compared with mice treated with BLM and sham, those treated with BLM, rmIL-31, and anti-IL-4RA mAb showed increased fibrosis of skin and lung, indicating that the pro-fibrotic effects of IL-31 were reduced but not completely prevented by anti-IL-4RA mAb. Similarly, anti-IL-4RA mAb significantly attenuated but did not completely prevent the enhanced expression of *Il4*, *Il6*, *Il10*, and *Tgfb1* in skin and lung of mice induced by rmIL-31 (Supplementary Fig. 7c). These results indicate that the effects of IL-31 on fibrosis and cytokine expression are partially but not fully mediated by signaling through IL-4RA. We also evaluated the contribution of TGF-β1 to IL-31-mediated fibrosis by administrating an anti-TGF-β1 mAb alongside with rmIL-31 and BLM. As shown in Supplementary Fig. 8a, b, the pro-fibrotic effects of IL-31 were suppressed but not completely prevented by anti-TGF-β1 mAb. As for cytokine expression, mice treated with BLM, rmIL-31, and anti-TGF-β1 mAb showed decreased expression of TGF-β1 compared with those treated with BLM and rmIL-31, but expression levels of *Il4*, *Il6*, *Il10*, *Il17a*, *Tnf*, or *Ifng* were not significantly affected by anti-TGF-β1 mAb (Supplementary Fig. 8c). These results suggest that the pro-fibrotic effects of IL-31 in BLM-SSc mice are at least partially mediated by the enhanced production of TGF-β1. Consistently, anti-TGF-β1 treatment diminished the collagen induction by IL-31 in DFs from SSc patients (Supplementary Fig. 9). Regarding the interaction of IL-31 and TGF-β1 in the context of BLM-induced fibrosis, in vitro experiments showed that anti-TGF-β1 mAb decreased *Il31* expression and increased *Il31ra* expression in fibroblasts from the lungs of BLM-SSc mice (Supplementary Fig. 10). The latter finding, which is in line with the study by Yaseen et al. ^34^, might appear to be inconsistent with the results that anti-TGF-β1 treatment decreased collagen induction by IL-31. Although further studies are needed to fully elucidate the interaction between IL-31 and TGF-β1 in the pathogenesis of SSc, one possibility is that IL-31RA expression in SSc patients and BLM model is already elevated (Figs. 2, 5f) and the further up-regulation by anti-TGF-β1 antibody does not necessarily lead to the enhanced signaling through the IL-31/IL-31RA axis. Overall, both TGF-β1 and signaling through IL-4RA are important but not sufficient for the pro-fibrotic effects of IL-31 in BLM-SSc mice.

### IL-31 promotes Th2 polarization in BLM-SSc mice

**Fig. 7:**
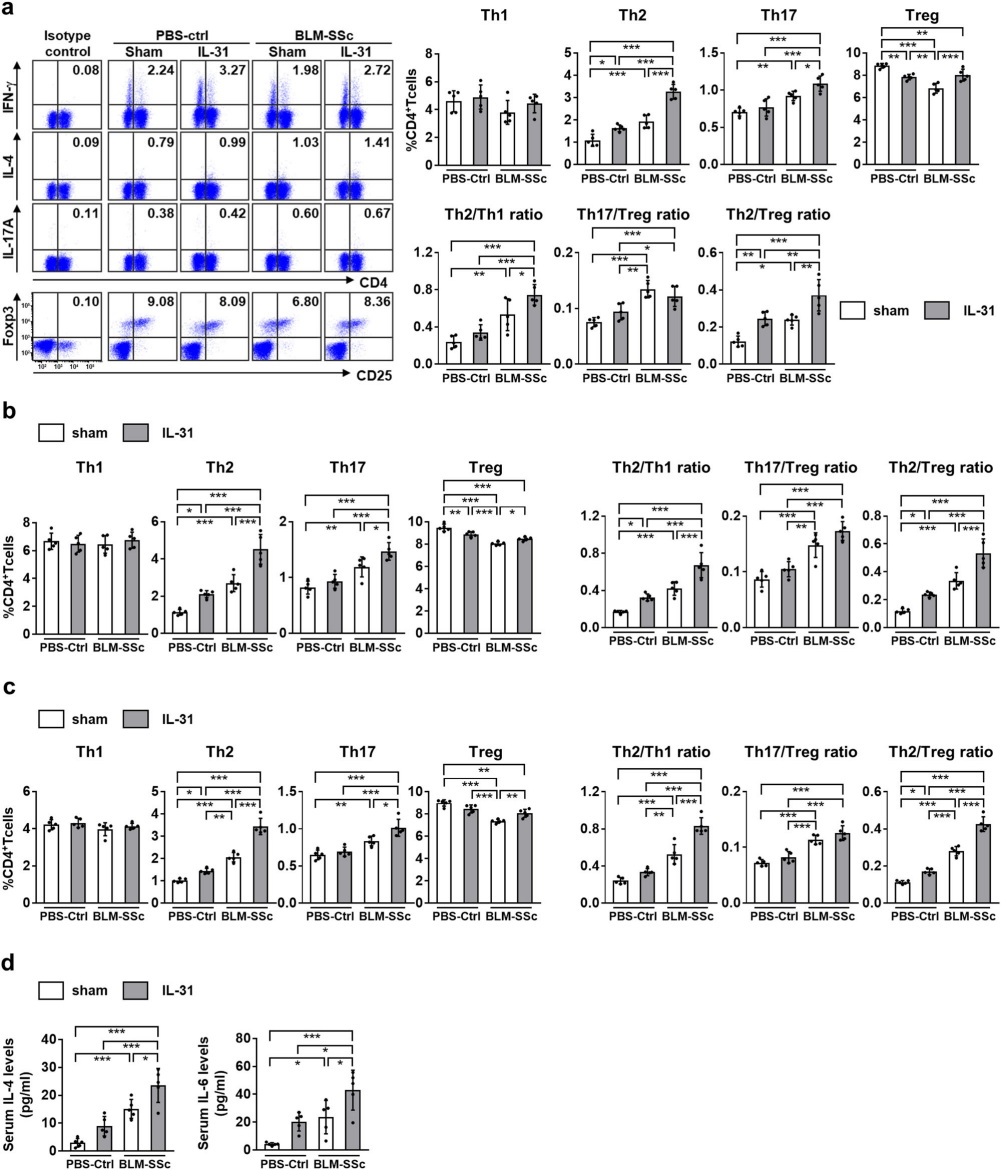
IL-31 enhanced Th2 polarization in BLM-SSc mice. **a**–**c** The frequencies of Th1, Th2, Th17, and Treg cells in BLM-SSc and PBS-treated control (PBS-Ctrl) mice treated with either rmIL-31 or sham were analyzed by flow cytometry in spleen (**a**), lung (**b**), and lung-draining lymph nodes (**c**). Exact *p* values (PBS-Ctrl + sham vs. PBS-Ctrl + IL-31, PBS-Ctrl + sham vs. BLM-SSc + sham, PBS-Ctrl + sham vs. BLM-SSc + IL-31, PBS-Ctrl + IL-31 vs. BLM-SSc + sham, PBS-Ctrl + IL-31 vs. BLM-SSc + IL-31, BLM-SSc + sham vs. BLM-SSc + IL-31) = 0.960, 0.417, 0.996, 0.145, 0.841, 0.625 (Th1, spleen); 0.017, 0.0002, 0.00000000007, 0.342, 0.00000001, 0.0000003 (Th2, spleen); 0.795, 0.005, 0.000006, 0.059, 0.00006, 0.041 (Th17, spleen); 0.001, 0.0000001, 0.008, 0.002, 0.926, 0.0003 (Treg, spleen); 0.521, 0.005, 0.00002, 0.079, 0.0002, 0.043 (Th2/Th1 ratio, spleen); 0.206, 0.00005, 0.0007, 0.003, 0.044, 0.521 (Th17/Treg ratio, spleen); 0.007, 0.011, 0.000004, 0.996, 0.006, 0.004 (Th2/Treg ratio, spleen); 0.966, 0.932, 0.996, 0.999, 0.904, 0.849 (Th1, lung); 0.018, 0.0003, 0.00000002, 0.233, 0.000003, 0.00009 (Th2, lung); 0.633, 0.005, 0.00002, 0.059, 0.0002, 0.039 (Th17, lung); 0.005, 0.0000002, 0.00002, 0.0002, 0.050, 0.048 (Treg, lung); 0.031, 0.0007, 0.0000002, 0.264, 0.00002, 0.0007 (Th2/Th1 ratio, lung); 0.363, 0.0002, 0.000003, 0.005, 0.00005, 0.125 (Th17/Treg ratio, lung); 0.034, 0.0002, 0.00000005, 0.082, 0.000004, 0.0005 (Th2/Treg ratio, lung); 0.939, 0.488, 0.961, 0.221, 0.719, 0.772 (Th1, lung-draining lymph nodes); 0.038, 0.000009, 0.00000000006, 0.002, 0.000000001, 0.0000003 (Th2, lung-draining lymph nodes); 0.839, 0.010, 0.00001, 0.051, 0.00005, 0.013 (Th17, lung-draining lymph nodes); 0.057, 0.000001, 0.001, 0.0002, 0.230, 0.009 (Treg, lung-draining lymph nodes); 0.225, 0.00008, 0.000000005, 0.004, 0.00000006, 0.00003 (Th2/Th1 ratio, lung-draining lymph nodes); 0.398, 0.00003, 0.000001, 0.0005, 0.00001, 0.244 (Th17/Treg ratio, lung-draining lymph nodes); 0.011, 0.00000007, 0.000000000007, 0.00002, 0.0000000002, 0.0000005 (Th2/Treg ratio, lung-draining lymph nodes). **d** Serum levels of IL-4 and IL-6 were assessed by ELISA. Exact *p* values (PBS-Ctrl + sham vs. PBS-Ctrl + IL-31, PBS-Ctrl + sham vs. BLM-SSc + sham, PBS-Ctrl + sham vs. BLM-SSc + IL-31, PBS-Ctrl + IL-31 vs. BLM-SSc + sham, PBS-Ctrl + IL-31 vs. BLM-SSc + IL-31, BLM-SSc + sham vs. BLM-SSc + IL-31) = 0.129, 0.001, 0.000002, 0.103, 0.0001, 0.019 (IL-4); 0.085, 0.032, 0.00007, 0.954, 0.012, 0.033 (IL-6). *n* = 5. Data are shown as mean ± SD. *p < 0.05, **p < 0.01, and ***p < 0.001. Significance was determined using one-way analysis of variance followed by Tukey’s post hoc comparison test. The results shown are representative of three independent experiments with similar results. PBS-Ctrl, PBS-treated control. Source data are provided as a Source Data file.

We next examined the effect of rmIL-31 on the differentiation of splenic T cells (Fig. 7a, Supplementary Fig. 11). BLM-SSc mice showed an increased frequency of Th2 and Th17 cells and a decreased frequency of Treg cells compared to PBS-treated control mice. Furthermore, the ratios of Th2/Th1, Th17/Treg, and Th2/Treg cells were increased in BLM-SSc mice, which is in accordance with the Th2 and Th17 polarization of SSc^10^. Treatment with rmIL-31 further enhanced the increased Th2 cell frequency, Th2/Th1 ratio, and Th2/Treg ratio in BLM-SSc mice. Although rmIL-31 significantly increased the frequency of Th17 cells in BLM-SSc mice, it also increased the frequency of Treg cells. Thus, rmIL-31 had no statistically significant effect on the Th17/Treg ratio in BLM-SSc mice. In PBS-control mice, administration of rmIL-31 induced a significant increase in Th2 cell frequency and a decrease in Treg cell frequency. Administration of rmIL-31 did not significantly change Th2/Th1 ratio or Th17/Treg ratio, but increased Th2/Treg ratio in PBS-control mice. Similar results were obtained on T cells in lung and lung-draining lymph nodes, where rmIL-31 increased the frequency of Th2 and Th17 cells, Th2/Th1 ratio, and Th2/Treg ratio in BLM-SSc mice (Fig. 7b, c). As for serum cytokine levels, rmIL-31 enhanced the overproduction of IL-4 and IL-6 in BLM-SSc mice (Fig. 7d). Thus, IL-31 promoted Th2 polarization in BLM-SSc mice. We also examined the effects of rmIL-31 on macrophage differentiation in spleen, but rmIL-31 did not significantly change M1 macrophage frequency, M2 macrophage frequency, or M2/M1 ratio (Supplementary Fig. 12).

### Anti-IL-31RA mAb reduces fibrosis and Th2 polarization in BLM-SSc mice

**Fig. 8:**
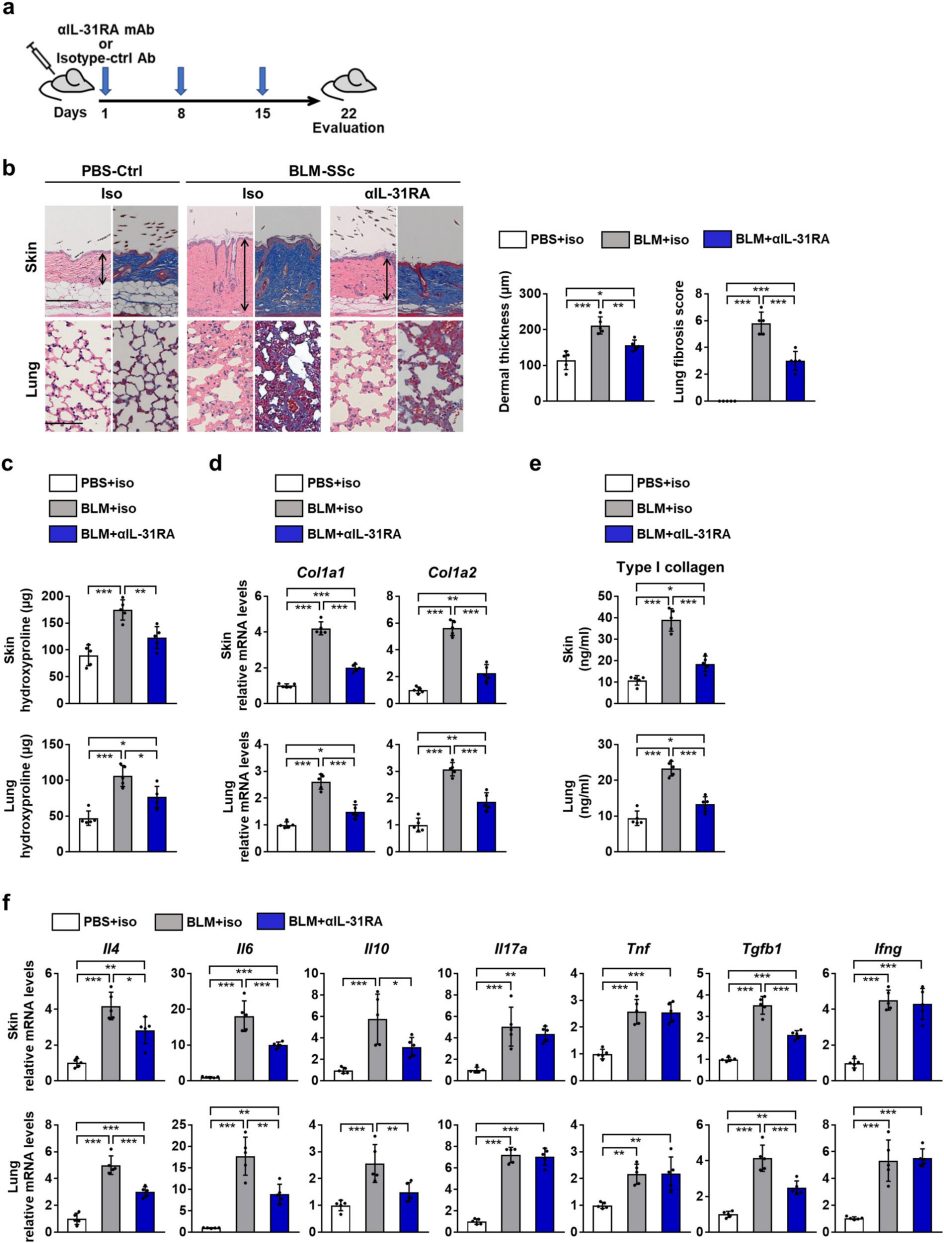
Anti-IL-31RA antibody attenuated fibrosis induced in BLM-SSc mice. **a** BLM-SSc and PBS-treated control (PBS-Ctrl) mice were administrated with anti-mouse IL-31RA antibody (αIL-31RA) or isotype control IgG (iso) on days 1, 8, and 15, and sacrificed on day 22 for evaluation. **b** Representative histological sections of the skin and lungs stained with hematoxylin and eosin (left) and Masson trichrome (right) were shown (horizontal scale bars=100 μm in skin; 20 µm in lung). Vertical bars with arrows represent dermal thickness. Dermal thickness and lung fibrosis score were assessed histologically. Exact p values (PBS-Ctrl + iso vs. BLM-SSc + iso, PBS-Ctrl + iso vs. BLM-SSc + αIL-31RA, BLM-SSc + iso vs. BLM-SSc + αIL-31RA) = 0.00004, 0.028, 0.004 (dermal thickness); 0.0000002, 0.00002, 0.00004 (lung fibrosis score). **c** Hydroxyproline contents in skin and lung samples. Exact *p* values (PBS-Ctrl + iso vs. BLM-SSc + iso, PBS-Ctrl + iso vs. BLM-SSc + αIL-31RA, BLM-SSc + iso vs. BLM-SSc + αIL-31RA) = 0.00005, 0.052, 0.003 (skin); 0.00004, 0.011, 0.012 (lung). **d** Relative mRNA expression levels of *Col1a1* and *Col1a2* in the skin and lungs were evaluated by real-time PCR. Exact *p* values (PBS-Ctrl + iso vs. BLM-SSc + iso, PBS-Ctrl + iso vs. BLM-SSc + αIL-31RA, BLM-SSc + iso vs. BLM-SSc + αIL-31RA) = 0.0000000003, 0.00009, 0.00000002 (*Co11a1*, skin); 0.00000002, 0.005, 0.0000006 (*Co11a2*, skin); 0.0000004, 0.014, 0.00002 (*Col1a1*, lung); 0.0000002, 0.001, 0.00005 (*Col1a2*, lung). Relative fold differences (BLM-SSc + iso, BLM-SSc + αIL-31RA) = 4.21, 2.00 (*Col1a1*, skin); 5.64, 2.27 (*Col1a2*, skin); 2.62, 1.50 (*Col1a1*, lung); 3.08, 1.86 (*Col1a2*, lung). **e** Protein expression of type I collagen in the skin and lungs was evaluated by ELISA. Exact p values (PBS-Ctrl + iso vs. BLM-SSc + iso, PBS-Ctrl + iso vs. BLM-SSc + αIL-31RA, BLM-SSc + iso vs. BLM-SSc + αIL-31RA) = 0.0000002, 0.021, 0.000007 (skin); 0.0000005, 0.026, 0.00002 (lung). **f** The mRNA expression of *Il4*, *Il6*, *Il10*, *Il17a*, *Tnf*, *Tgfb1*, and *Ifng* in the skin and lungs of these mice was evaluated by real-time PCR. Exact p values (PBS-Ctrl + iso vs. BLM-SSc + iso, PBS-Ctrl + iso vs. BLM-SSc + αIL-31RA, BLM-SSc + iso vs. BLM-SSc + αIL-31RA) = 0.00001, 0.002, 0.015 (*Il4*, skin); 0.0000005, 0.0003, 0.0007 (*Il6*, skin); 0.0006, 0.083, 0.037 (*Il10*, skin); 0.0003, 0.001, 0.625 (*Il17a*, skin); 0.00004, 0.00005, 0.996 (*Tnf*, skin); 0.00000001, 0.00006, 0.00001 (*Tgfb1*, skin); 0.000003, 0.000005, 0.848 (*Ifng*, skin); 0.0000001, 0.0001, 0.0001 (*Il4*, lung); 0.000003, 0.003, 0.001 (*Il6*, lung); 0.0005, 0.251, 0.010 (*Il10*, lung); 0.000000007, 0.000000009, 0.927 (*Il17a*, lung); 0.003, 0.002, 0.997 (*Tnf*, lung); 0.0000009, 0.001, 0.0005 (*Tgfb1*, lung); 0.00004, 0.00002, 0.943 (*Ifng*, lung). Relative fold differences (BLM-SSc + iso, BLM-SSc + αIL-31RA) = 4.19, 2.83 (*Il4*, skin); 18.09, 10.01 (*Il6*, skin); 5.75, 3.15 (*Il10*, skin); 5.05, 4.38 (*Il17a*, skin); 2.57, 2.55 (*Tnf*, skin); 3.52, 2.14 (*Tgfb1*, skin); 4.51, 4.30 (*Ifng*, skin); 5.00, 3.01 (*Il4*, lung); 17.72, 8.92 (*Il6*, lung); 2.56, 1.50 (*Il10*, lung); 7.20, 7.05 (*Il17a*, lung); 2.17, 2.19 (*Tnf*, lung); 4.14, 2.48 (*Tgfb1*, lung); 5.34, 5.54 (*Ifng*, lung). *n* = 5. Data are shown as mean ± SD. *p < 0.05, **p < 0.01, and ***p < 0.001. One-way analysis of variance followed by Tukey’s post hoc comparison test was used. The results shown are representative of three independent experiments with similar results. PBS-Ctrl, PBS-treated control; αIL-31RA, anti-mouse IL-31RA antibody; iso, isotype control IgG. Source data are provided as a Source Data file.

**Fig. 9:**
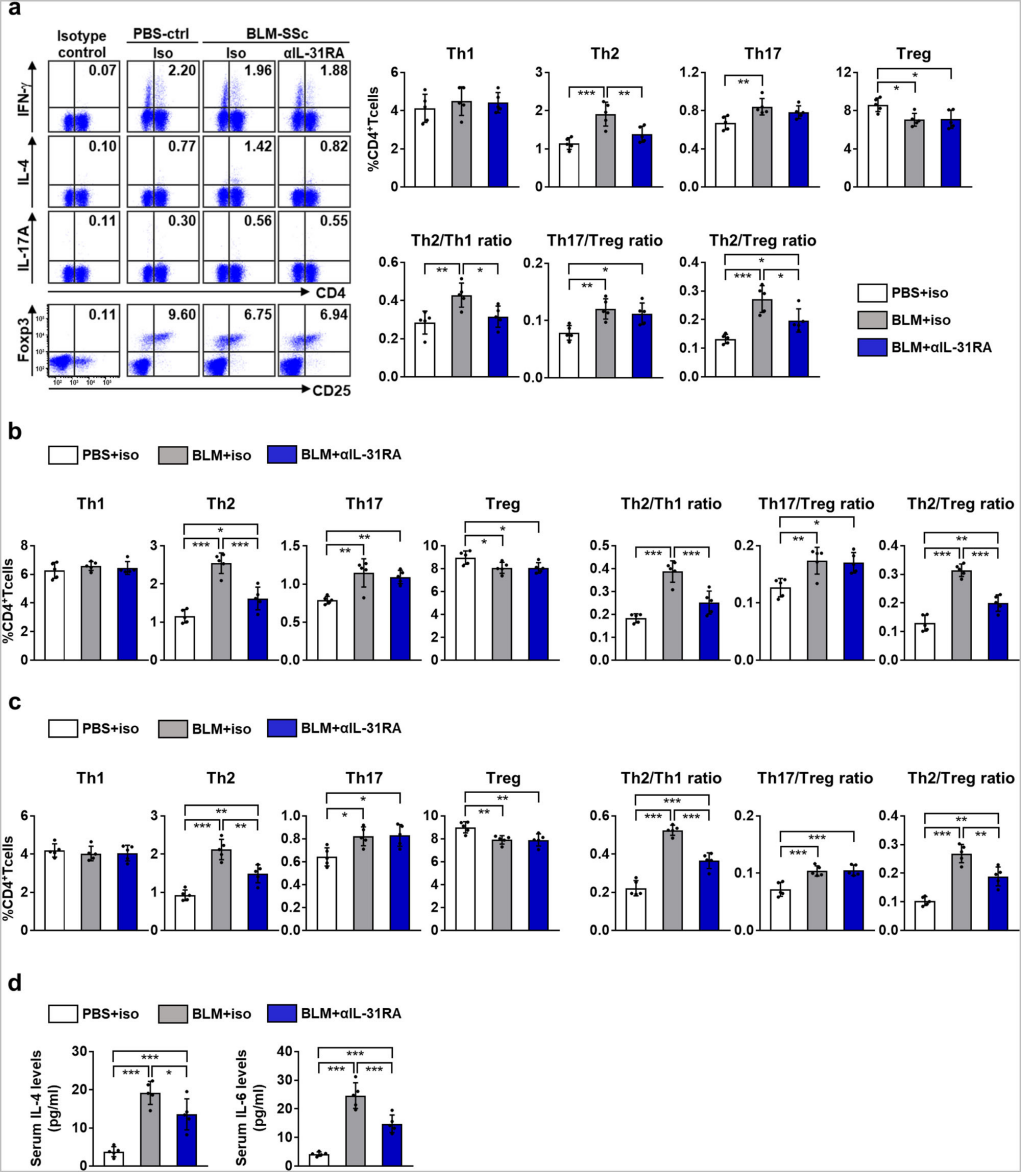
Anti-IL-31RA antibody attenuated Th2 polarization in BLM-SSc mice. **a**–**c** The frequencies of Th1, Th2, Th17, and Treg cells in BLM-SSc and PBS-treated control (PBS-Ctrl) mice administrated with anti-mouse IL-31RA antibody (αIL-31RA) or isotype control IgG (iso) were analyzed by flow cytometry in spleen (**a**), lung (**b**), and lung-draining lymph nodes (**c**). Exact *p* values (PBS-Ctrl + iso vs. BLM-SSc + iso, PBS-Ctrl + iso vs. BLM-SSc + αIL-31RA, BLM-SSc + iso vs. BLM-SSc + αIL-31RA) = 0.649, 0.769, 0.978 (Th1, spleen); 0.0006, 0.269, 0.010 (Th2, spleen); 0.010, 0.085, 0.463 (Th17, spleen); 0.021, 0.026, 0.994 (Treg, spleen); 0.006, 0.674, 0.027 (Th2/Th1 ratio, spleen); 0.005, 0.020, 0.712 (Th17/Treg ratio, spleen); 0.0002, 0.042, 0.019 (Th2/Treg ratio, spleen); 0.547, 0.830, 0.878 (Th1, lung); 0.000004, 0.032, 0.0002 (Th2, lung); 0.002, 0.005, 0.768 (Th17, lung); 0.042, 0.040, 0.999 (Treg, lung); 0.00001, 0.052, 0.0006 (Th2/Th1 ratio, lung); 0.006, 0.010, 0.957 (Th17/Treg ratio, lung); 0.0000003, 0.003, 0.00004 (Th2/Treg ratio, lung); 0.785, 0.830, 0.996 (Th1, lung-draining lymph nodes); 0.000005, 0.004, 0.002 (Th2, lung-draining lymph nodes); 0.015, 0.012, 0.988 (Th17, lung-draining lymph nodes); 0.008, 0.007, 0.999 (Treg, lung-draining lymph nodes); 0.00000005, 0.0001, 0.00005 (Th2/Th1 ratio, lung-draining lymph nodes); 0.0008, 0.0006, 0.983 (Th17/Treg ratio, lung-draining lymph nodes); 0.000002, 0.001, 0.002 (Th2/Treg ratio, lung-draining lymph nodes). **d** Serum levels of IL-4 and IL-6 were assessed by ELISA. Exact *p* values (PBS-Ctrl + iso vs. BLM-SSc + iso, PBS-Ctrl + iso vs. BLM-SSc + αIL-31RA, BLM-SSc + iso vs. BLM-SSc + αIL-31RA) = 0.00001, 0.0007, 0.031 (IL-4); 0.0000008, 0.0005, 0.0009 (IL-6). *n* = 5. Data are shown as mean ± SD. *p < 0.05, **p < 0.01, and ***p < 0.001. One-way analysis of variance followed by Tukey’s post hoc comparison test was used. The results shown are representative of three independent experiments with similar results. PBS-Ctrl, PBS-treated control; αIL-31RA, anti-mouse IL-31RA antibody; iso, isotype control IgG. Source data are provided as a Source Data file.

To explore the role of IL-31 in the development of SSc, we assessed fibrosis and Th2 polarization of BLM-SSc mice treated with functionally blocking anti-IL-31RA mAb (NRA0049). We administered anti-IL-31RA mAb weekly along with daily injections of BLM for three weeks (Fig. 8a). Anti-IL-31RA mAb significantly reduced dermal thickness, lung fibrosis score, and collagen content in the skin and lungs of BLM-SSc mice compared with isotype control IgG (Fig. 8b–e). Furthermore, anti-IL-31RA mAb attenuated the expression of IL-4, IL-6, IL-10, and TGF-β1 that was up-regulated in the skin and lungs of BLM-SSc mice (Fig. 8f, Supplementary Fig. 13). Treatment with anti-IL-31RA mAb also significantly reduced Th2 cell frequency, Th2/Th1 ratio, and Th2/Treg ratio, but not Th17/Treg ratio in spleen, lung, and lung-draining lymph nodes of BLM-SSc mice (Fig. 9a–c). Furthermore, overproduction of IL-4 and IL-6 in the sera of BLM-SSc mice was ameliorated by anti-IL-31RA mAb (Fig. 9d). Overall, anti-IL-31RA mAb significantly attenuated BLM-induced fibrosis and Th2 polarization.

## Discussion

In this study, we showed the efficacy of anti-IL-31RA mAb in a murine model of SSc. Although previous studies have suggested pro-fibrotic effects of IL-31^34,44^, the efficacy of anti-IL-31RA mAb has not been explored in SSc. Here, we initially demonstrated that serum IL-31 levels were significantly elevated in SSc patients (Fig. 1a). Of note, serum IL-31 levels positively correlated with the severity of skin and lung fibrosis and serum levels of IL-4, IL-6, and IL-13 (Fig. 1b and Table 1), which suggests the association of IL-31 with fibrosis and Th2 immune responses in SSc. Moreover, both IL-31 and IL-31RA expression was significantly increased in DFs from SSc patients compared with those from healthy controls (Fig. 2). In SSc DFs, IL-31 induced collagen production and the expression of cytokines that promote fibrosis and Th2 polarization (Fig. 3). Importantly, these effects were mainly mediated by IL-31RA and STAT3 (Fig. 4). In BLM-SSc mice, exogenous administration of rmIL-31 resulted in exacerbated skin and lung fibrosis, increased proportion of Th2 cells, and elevated expression of the cytokines associated with fibrosis and Th2 responses (Figs. 5–7). These results led us to propose that IL-31 augments fibrosis and Th2 polarization in SSc. Furthermore, anti-IL-31RA mAb significantly attenuated fibrosis and Th2 polarization in BLM-SSc mice (Figs. 8, 9), demonstrating the crucial role of IL-31 in the development of SSc. Overall, in keeping with the recent article by Yaseen et al. ^34^, these results suggest that IL-31 is a promising therapeutic target for SSc.

Excessive extracellular matrix production by fibroblasts is an important pathological feature of SSc that leads to progressive fibrosis of the skin and internal organs^45^. Recently, Yaseen et al. have shown the pro-fibrotic effects of IL-31 in both in vitro and in vivo^34^. Consistent with their results, we showed that IL-31 promoted excessive collagen production both in SSc DFs and BLM-SSc mice. Further, we also demonstrated that IL-31 promoted SSc-like phenotype in SSc DFs including increased differentiation to myofibroblasts (Fig. 3).

Th2 polarization is a major immunological abnormality in SSc that is observed both in the patients and animal models of SSc^3^. Accumulating evidence has shown that Th2 cells are the main producers of IL-31^19,20^. A recent study has also reported that IL-4 is essential for Th2 cells to secrete IL-31^46^. Together with the up-regulation of IL-31 and IL-31RA by Th2 cytokines (Fig. 2e), the overexpression of IL-31 in SSc patients presumably reflects the Th2 dominance of the disease. Furthermore, IL-31 enhanced the expression of IL-6, IL-33, and CCL2 in SSc DFs (Fig. 3i, j). Because these cytokines have been shown to promote Th2 cytokine expression in different cells and tissues, this result suggests that IL-31 promotes Th2 cytokine expression in SSc by up-regulating these pro-Th2 cytokines in DFs^40–42^. Indeed, in vivo studies showed that IL-31 promoted Th2 polarization in BLM-SSc mice (Figs. 6, 7). Since IL-31RA blocking mAb attenuated the expression of Th2 cytokines and the differentiation of Th2 cells in BLM-SSc mice (Figs. 8, 9), the results of this study indicate that IL-31 potentiates Th2 polarization in SSc.

SSc is one of the most devastating autoimmune diseases with high morbidity and mortality, which is largely attributed to the lack of effective treatment. Hence, developments of new drugs are highly desired in the treatment of SSc. The current study suggests that IL-31 contributes to the development of SSc in two ways: directly inducing collagen production in fibroblasts, and indirectly promoting Th2 polarization by inducing pro-Th2 cytokines in fibroblasts. Th2 polarization then further enhances IL-31 production, which forms a vicious cycle via the IL-31/IL-31RA axis, leading to the development of SSc. Although further studies are required to clarify the exact role of IL-31 in the pathogenesis of SSc, our study offered a previously unidentified insight into the role of IL-31 in SSc. Moreover, our study provides the evidence that anti-IL-31RA mAb significantly ameliorates fibrosis and Th2 polarization in an experimental model of SSc. These results may support the clinical translation of anti-IL-31RA mAb for the treatment of SSc patients.

## Methods

### Serum samples

Serum samples for ELISA to determine IL-4, IL-6, IL-13, and IL-31 levels were obtained from 74 Japanese SSc patients (67 women and seven men; mean ± SD age, 51 ± 17 years; disease duration, 2.0 ± 2.2 years). All patients fulfilled the ACR/EULAR classification criteria for SSc^47^. Patients treated with corticosteroids or other immunosuppressants prior to their first visits were excluded. Disease duration was calculated as the interval from the onset of the first SSc clinical manifestations other than Raynaud’s phenomenon to serum sampling. Patients were categorized by LeRoy’s classification system:^48^ 55 dcSSc patients (49 women and six men; age, 50 ± 16 years; disease duration, 2.1 ± 2.2 years) and 19 lcSSc patients (18 women and one man; age, 54 ± 19 years; disease duration, 1.6 ± 2.0 years). As controls, 14 age- and sex-matched healthy Japanese individuals were enrolled. The whole study was conducted in compliance with the Declaration of Helsinki and approved by the ethics committee of the University of Tokyo Graduate School of Medicine. Written informed consent was obtained from all participants.

### Clinical assessment

Complete medical histories, physical examinations, and laboratory tests, including measurements of %VC and %DLco were performed in all patients. %VC values less than 80% and %DLco values less than 75% were considered abnormal. Skin thickness was assessed by MRSS^35^. Organ involvement was defined as follows:^49^ pulmonary fibrosis = bibasilar fibrosis on chest radiography and high-resolution computed tomography; pulmonary hypertension = right ventricular systolic pressure of 35 mmHg or more on echocardiogram; esophagus = distal esophageal hypomotility on barium-contrast radiography and gastroesophageal reflux disease detected at upper gastrointestinal endoscopy; kidneys = malignant hypertension and rapidly progressive renal failure.

### Mice and experimental protocol

Wild-type C57BL/6 mice were purchased from The Jackson Laboratory (Bar Harbor, ME, USA) and housed under controlled temperature (21 ± 1˚C) and relative humidity (60 ± 10%) with a 12/12 h dark/light cycle. BLM (Nippon Kayaku, Tokyo, Japan) was dissolved in PBS at a concentration of 1 mg/ml. To generate BLM-SSc mice, BLM was injected at a dose of 200 μg subcutaneously into the shaved backs of female mice with a 26-gauge needle daily^50^. The mice treated with PBS instead of BLM were used as controls for BLM-SSc mice. To evaluate the effects of IL-31 on BLM-SSc mice, rmIL-31 (200 ng; Abcam, Cambridge, MA, USA) and BLM was injected subcutaneously daily for 2 weeks (day 1 to day 14). Saline was used as a sham for rmIL-31. To evaluate the effects of blocking IL-31 signaling, the functionally blocking anti-mouse IL-31RA mAb (NRA0049) or mouse IgG1 kappa isotype control (eBioscience, San Diego, CA, USA) was injected at a dose of 200 μg intraperitoneally every 7th day (day 1, 8, and 15), with daily administration of BLM for 3 weeks (day 1 to day 21). NRA0049 was kindly provided by Chugai Pharmaceutical Co., Ltd (Tokyo, Japan). To evaluate the preventive effects of blocking the signaling through IL-4RA in the pro-fibrotic responses induced by rmIL-31, anti-IL-4RA mAb (BD Biosciences, San Diego, CA, USA) was injected at a dose of 200 µg subcutaneously twice weekly (day 1, 4, 8, 11), alongside with daily administration of rmIL-31 and BLM for 2 weeks (day 1 to day 14, respectively). To evaluate the preventive effects of blocking TGF-β1 signaling in the pro-fibrotic responses induced by rmIL-31, anti-TGF-β1 mAb (R&D Systems, Minneapolis, MN, USA) was injected at a dose of 200 µg subcutaneously twice weekly (day 1, 4, 8, 11), alongside with daily administration of rmIL-31 and BLM for 2 weeks (day 1 to day 14, respectively). All mice used in the study were six weeks old. Five mice per group were examined. All studies involving mice strictly adhered to the guidelines for animal experiments of the University of Tokyo and were approved by the Committee on Animal Experimentation of the University of Tokyo Graduate School of Medicine.

### Production of anti-IL-31RA mAb

Anti-IL-31RA mAb (NRA0049) was produced by Chugai Pharmaceutical Co., Ltd. in the following way. Three New Zealand white rabbits (Kitayama Labes, Kyoto, Japan) were immunized four times with mouse IL-31RA (smNR10-FLAG). The animals were then euthanized and their peripheral blood mononuclear cells (PBMCs) and splenocytes were harvested. All animal care and experimental protocols were performed in accordance with the guidelines for the care and use of laboratory animals at Chugai Pharmaceutical Co., Ltd. The protocol was approved by the Institutional Animal Care and Use Committee at Chugai Pharmaceutical Co., Ltd.

### Antibody screening using flow cytometry

Anti-IL-31RA antibody-producing B cells were obtained using the method described by Seeber *et al*. ^51^. The PBMCs and splenocytes collected after sensitization with mouse IL-31RA (smNR10-FLAG) were labeled with PE-conjugated mouse anti-rabbit IgG mAb (Southern Biotech, Birmingham, AL, USA) and anti-mouse IgG microbeads (Miltenyi Biotec, Gladbach, Germany), and were enriched using magnetic-activated cell sorting (MACS). The cells were then labeled with biotinylated smNR10-FLAG and FITC-streptavidin (Miltenyi Biotec), and the FITC- and PE-positive cells were collected using a FACS Aria III sorter (BD Biosciences). Next, the collected cells were seeded in a microtiter plate at a density of one cell/well, and were incubated for one week. These culture supernatants were screened for mouse IL-31RA-recognizing clones using FACS with DG44 cells stained with CellTrace Far Red (Thermo Fisher Scientific, San Jose, CA, USA) and unstained DG44 cells expressing mouse IL-31RA (MNT24), and the genes encoding antibody variable regions of B cells from 352 of these wells were cloned. These DNA fragments encoding the rabbit immunoglobulin variable regions were inserted into an expression vector containing mouse IgG1/kappa chain constant regions to force their expression as recombinant IgG in Expi293 cells (Thermo Fisher Scientific). The recombinant IgG was then purified and isolated from the Expi293 cell culture supernatant using MabSelect SuRe LX-Sepharose (GE Healthcare, Waukesha, WI, USA).

### ELISA

ELISA was performed to evaluate the protein expression in serum, tissue homogenates,　cell lysates, and cell supernatants^52^. Human samples were analyzed using the specific ELISA kits for IL-4 (D4050, R&D Systems), IL-6 (D6050, R&D Systems), IL-13 (D1300B, R&D Systems), TGF-β1 (DB100B, R&D Systems), CTGF (DY9190-05, R&D Systems), MMP-9 (DMP900, R&D Systems), IL-33 (D3300B, R&D Systems), CCL2 (DCP00, R&D Systems), IL-31 (ab119546, abcam), MMP-1 (ab215083, abcam), IL-31RA (MBS923904, MyBioSource, San Diego, CA, USA), and type I collagen (EC1-E205, Adjusted Cell Experiment Laboratory, Kanagawa, Japan). Mouse samples were analyzed using the specific ELISA kits for IL-4 (M4000B, R&D Systems), IL-6 (M6000B, R&D Systems), IL-10 (M1000B, R&D Systems), TNF (MTA00B, R&D Systems), TGF-β1 (MB100B, R&D Systems), IFN-γ (MIF00, R&D Systems), IL-17A (ab216167, abcam), CTGF (LS-F26114-1, LifeSpan BioSciences, Seattle, WA, USA), IL-31 (LS-F5987, LifeSpan BioSciences), IL-31RA (LS-F14320, LifeSpan BioSciences), and type I collagen (NBP2-75822, Novus Biologicals, Littleton, CO, USA). To activate latent TGF-β1 to the immunoreactive form, acid activation and neutralization were performed before the assay of TGF-β1, as recommended by the manufacturer. All experiments were performed in accordance with the manufacturers’ instructions.

### Histological analysis

Skin and lung tissues were formalin-fixed, embedded in paraffin, and stained with hematoxylin and eosin. Dermal thickness, defined as the distance between the epidermal-dermal junction and the dermal-adipose junction, was measured. The severity of lung fibrosis was semi-quantitatively assessed, as described by Ashcroft et al. ^53^. Briefly, the grading criteria were as follows: grade 0 = normal lung; grade 1 = minimal fibrous thickening of alveolar or bronchiolar walls; grade 3 = moderate thickening of walls without obvious damage to lung architecture; grade 5 = increased fibrosis with definite damage to lung structure and formation of fibrous bands or small fibrous masses; grade 7 = severe distortion of the structure and large fibrous areas; and grade 8 = total fibrous obliteration of fields. Grades 2, 4, and 6 were used as intermediate pictures between the aforementioned criteria. Formalin-fixed, paraffin-embedded skin and lung tissues were also stained with Masson’s trichrome to evaluate the deposition of collagen. Each section was examined independently by two investigators (A.K. and A.Y.) in a blinded manner.

### Hydroxyproline assay

The collagen content of skin and lung tissue in mice was determined by QuickZyme Total Collagen Assay (QZBTOTCOL1, QuickZyme Biosciences, Leiden, The Netherlands) according to the manufacturer’s instructions. Total left lungs and 6-mm punch biopsy skin samples were used.

### RNA isolation and real-time PCR

Total RNA was isolated from tissues and cells using RNeasy spin columns (Qiagen, Crawley, UK) and ISOGEN II (Nippon Gene, Toyama, Japan), respectively, according to the manufacturers’ instructions. Total RNA from each sample was reverse-transcribed into cDNA. Gene expression was quantified using SYBR green real-time PCR master mix (TOYOBO, Osaka, Japan) and analyzed on an ABI Prism 7000 sequence detector (Applied Biosystems, Foster City, CA, USA). To normalize the amounts of loaded cDNA, GAPDH was used as an internal control. Relative fold differences were calculated using the comparative Ct method^50^. The sequences of the primers are described in Supplementary Table 1.

### Flow cytometry

Single-cell suspensions from spleen and lung-draining lymph nodes were prepared by gentle teasing. Subsequently, erythrocytes in spleen cell suspensions were lysed with ammonium chloride (StemCell Technologies, Cambridge, MA, USA). Lung cell suspensions were prepared by incubating minced lung in RPMI1640 medium with Liberase TM and DNase I (Roche, Indianapolis, IN, USA) for 30 min, followed by the lysis of erythrocytes with ammonium chloride^54^. For detection of Th1, Th2, or Th17 cells, single-cell suspensions from spleen, lungs, and lung-draining lymph nodes were prepared at 2 × 10^6^ /ml in RPMI 1640 medium and were stimulated with 25 ng/ml PMA (Adipogen, San Diego, CA, USA) and 1 mg/ml ionomycin (Sigma, St Louis, MO, USA) in the presence of 2 μM Monensin (Invitrogen, Carlsbad, CA, USA) for 6 h at 37 ˚C in 5% CO_2_. Samples were then surface stained with anti-CD3-PE mAb and anti-CD4-FITC mAb (eBioscience). After fixation and permeabilization with Cytofix/Cytoperm buffer (BD PharMingen, San Diego, CA, USA), samples were intracellularly stained with APC-conjugated mAbs against IFN-γ, IL-4, or IL-17A (eBioscience) for detection of Th1, Th2 or Th17 cells, respectively. APC-conjugated isotype-matched mAbs (eBioscience) were used as controls. For detection of Treg cells, the Mouse Regulatory T Cell Staining Kit (88-8118-40, eBioscience) was used as the manufacturer’s protocol. Briefly, single-cell suspensions from spleen, lungs, and lung-draining lymph nodes were surface stained with anti-CD3-PE/Cy7 mAb, anti-CD4-FITC mAb, and anti-CD25-APC mAb (eBioscience), followed by fixation and permeabilization with Cytofix/Cytoperm and intracellular staining with anti-Foxp3-PE mAb or rat IgG2a-PE (eBioscience) as a control. To evaluate M1 and M2 macrophage differentiation, single-cell suspensions from spleen were surface stained with anti-F4/80-FITC mAb, anti-CD11b-APC mAb, anti-CD206-PE mAb, and anti-CD11c-PE/Cy7 mAb (eBioscience). The antibodies used in this study are summarized in Supplementary Table 2. Samples were analyzed with a FACS Verse flow cytometer (BD Biosciences) and Kaluza software.

### Cell culture

Human DFs were obtained by skin biopsy from forearms of five dcSSc patients (four women and one man; age, 38 ± 12 years; disease duration, 1.4 ± 0.8 years) and age- and sex-matched five healthy individuals^55^. Human DFs were cultured in DMEM medium containing 10% FBS and antibiotic-antimycotic solution at 37 ˚C in 5% CO_2_. In some experiments, human DFs were serum starved for 12 h and then cultured for 24 h with rhIL-31, rhIL-4, or rhIL-13 (R&D Systems). In other experiments, human DFs were incubated with Fludarabine (50 µM; R&D Systems), Stattic (5 µM; R&D Systems), and CAS 285986-31-4 (50 µM; Sigma-Aldrich, Steinheim, Germany) for 24 h to inhibit the activation of STAT1, STAT3, and STAT5, respectively. Anti-TGF-β1 antibody (5 µg/ml; R&D Systems) was used to block TGF-β1 signaling in human DFs. Mouse lung fibroblasts were isolated by mincing and enzymatic digestion^56^. To obtain B cells and CD4^+^ T cells, cell suspensions were stained with biotinylated antibody mixture for negative selection (BD Biosciences), including CD4, CD43, and TER-119 for the isolation of B cells, and CD8a, CD11b, CD45R/B220, CD49b, and TER-119 for the isolation of CD4^+^ T cells. These cells were subsequently stained with magnetic particles conjugated with streptavidin (Miltenyi Biotec), and targeted cells were isolated using BD Imag Cell Separation System (BD Biosciences) according to the manufacturer’s protocol. To obtain macrophages, endothelial cells, and epithelial cells, anti-CD11b magnetic particles (BD Biosciences), anti-CD31 microbeads (Miltenyi Biotec), and EasySep Mouse Epithelial Cell Enrichment Kit II (ST-19868, Stem Cell Technologies) were used respectively, following the manufacturers’ instructions. Anti-TGF-β1 antibody (10 µg/ml; R&D Systems) was used to block TGF-β1 signaling in mouse fibroblasts.

### BrdU cell proliferation assay

Proliferation of cultured human DFs was evaluated by a BrdU cell proliferation ELISA kit (11647229001, Roche), following the manufacturer’s protocol. Briefly, DFs were incubated with rhIL-31 (50 ng/ml) or media alone for 24 h and treated with BrdU labeling solution for another 24 h. BrdU incorporation was detected colorimetrically using peroxidase-conjugated anti-BrdU antibody and tetramethylbenzidine substrate. The absorbance at 450 nm was measured.

### Apoptosis assay

Human DFs were incubated with rhIL-31 (50 ng/ml) or media alone for 48 h and stained with Annexin V-FITC and 7-AAD-PerCP-Cy5.5 (BioLegend), according to the manufacturer’s protocol. Samples were analyzed with a FACS Verse flow cytometer (BD Biosciences). Annexin-V^+^, 7-AAD^−^ cells were considered early apoptotic cells, and Annexin-V^+^, 7-AAD^+^ cells were considered late apoptotic cells, respectively^57^.

### Scratch migration assay

Human DFs were cultured to 100% confluency and serum-starved overnight. Scratch wounds were created in confluent monolayers using a sterile p200 pipette tip^58^. To block cell proliferation, DFs were pretreated with mitomycin C (10 µg/ml; Sigma-Aldrich), which was applied to DFs two hours before the scratch wounding and removed by three washes with PBS. After the scratch, DFs were stimulated with rhIL-31 or left untreated for 24 h. Wound closure was expressed as the percentage of wound reduction from the original wound. Wound area was measured using ImageJ software (National Institutes of Health, Bethesda, MD, USA).

### Immunofluorescence staining

Immunofluorescence staining was performed on formalin-fixed, paraffin-embedded skin sections obtained from the forearm of eight dcSSc patients, eight lcSSc patients, and six healthy controls. Rabbit anti-IL-31 antibody (abcam), rabbit anti-IL-31RA antibody (abcam), goat anti-FSP-1 antibody (GeneTex Irvine, CA, USA), and mouse anti-CD4 antibody (invitrogen) were used as primary antibodies. Alexa Flour 594-conjugated donkey anti-rabbit IgG antibody, Alexa Fluor 488-conjugated donkey anti-goat IgG antibody, and Alexa Flour 488-conjugated donkey anti-mouse IgG antibody were used as secondary antibodies. Sections were mounted with Vectashield with DAPI (Vector Laboratories, Burlingame, CA, USA). Images were taken by a Bio Zero BZ-8000 microscope (Keyence, Osaka, Japan). Each section was examined independently by two investigators (A.K. and A.Y.) in a blinded manner.

### Western blotting

Proteins were extracted from human DFs using RIPA lysis buffer (Santa Cruz Biotechnology, Santa Cruz, CA, USA). Extracted proteins were separated by SDS-PAGE and transferred to PVDF membranes (Millipore, Bedford, MA, USA). Membranes were blocked with PBS with 5% non-fat milk or 5% BSA in PBS/0.05% Tween 20 for one hour at room temperature and incubated with primary antibodies against STAT1, STAT3, STAT5, phosphorylated STAT1, phosphorylated STAT3, phosphorylated STAT5, α-SMA, and β-actin (Cell Signaling Technology) overnight at 4 ˚C, followed by incubation with HRP-conjugated secondary antibodies (Cell Signaling Technology) for one hour at room temperature. Proteins were visualized using Chemi-Lumi One L (Nacalai Tesque, Kyoto, Japan). Density of the bands was quantified using ImageJ 1.53e software (National Institutes of Health).

### RNA interference

Cells were transfected with IL-31 siRNA, IL-31RA siRNA, OSMR siRNA, or control scrambled siRNA (Santa Cruz Biotechnology) for 24 h using HiPerFect Transfection Reagent (Qiagen) according to the manufacturer’s protocol. Then the cells were serum starved for 24 h and used for further studies.

### ChIP assay

ChIP assay was performed using EpiQuik ChIP kit (P-2002-3, Epigentek, Farmingdale, NY, USA) according to the manufacturer’s instruction. Briefly, human DFs were cross-linked with 1% formaldehyde and the cross-linked chromatin was sonicated to an average size of 300–500 base pairs. Anti-phosphorylated STAT3 antibody (Santa Cruz Biotechnology) was used for immunoprecipitation with normal rabbit IgG (Santa Cruz Biotechnology) as the negative control. The immunoprecipitated chromatin was analyzed by agarose gel electrophoresis and quantitative real-time PCR using primers specific for the HS4 region:^43^ 5′- TTCACATGAGCATTTGAGTGTATTG -3′ and 5′-TCGTCAGTGTGTAACCCTCATC -3′.

### Statistical analysis

Data are expressed as means ± SD. Statistical analysis was performed by two-tailed Mann-Whitney *U* test for two-group comparison, Fisher’s exact probability test for frequency analysis, and Spearman’s rank correlation coefficient for clinical correlation. One-way analysis of variance followed by Tukey’s post hoc comparison test was used for multiple group comparison. P values less than 0.05 were considered significant. All analyses were performed using GraphPad Prism 7.03 (GraphPad Software, La Jolla, CA, USA).

### Reporting summary

Further information on research design is available in the Nature Research Reporting Summary linked to this article.

## Supplementary information

Supplementary Information

Reporting Summary

## Data Availability

All data generated or analyzed during this study are available within the article or supplementary information or from the corresponding author upon reasonable request. Source data are provided with this paper.

## Source data

Source Data
