# Supplementary material for: Interleukin-31 promotes fibrosis and T helper 2 polarization in systemic sclerosis
Source: Nat Commun. 2021 Oct 12;12:5947. doi: 10.1038/s41467-021-26099-w (PMC8511151; doi:10.1038/s41467-021-26099-w)

Supplementary Information

Interleukin-31 promotes fibrosis and T helper 2 polarization in systemic sclerosis

Ai Kuzumi[1], Ayumi Yoshizaki[1*], Kazuki M Matsuda[1], Hirohito Kotani[1], Yuta Norimatsu[1], Maiko Fukayama[1], Satoshi Ebata[1], Takemichi Fukasawa[1], Asako Yoshizaki-Ogawa[1], Yoshihide Asano[1], Kyojiro Morikawa[2], Yutaka Kazoe[3], Kazuma Mawatari[2], Takehiko Kitamori[4], Shinichi Sato[1*]

[1] Department of Dermatology, Graduate School of Medicine, The University of Tokyo, Tokyo, Japan.

[2] Department of Applied Chemistry, Graduate School of Engineering, The University of Tokyo, Tokyo, Japan.

[3] Department of System Design Engineering, Faculty of Science and Technology, Keio University, Yokohama, Japan.

[4] Department of Bioengineering, Graduate School of Engineering, The University of Tokyo, Tokyo, Japan.

[*]Correspondence and reprint requests to:

Ayumi Yoshizaki, M.D., Ph.D. and Shinichi Sato, M.D., Ph.D.

Department of Dermatology, Graduate School of Medicine, The University of Tokyo, 7-3-1 Hongo, Bunkyo-ku, Tokyo 113-8655, Japan.

Telephone: +81-3-5800-8661, Fax: +81-3-3814-1503

E-mail: ayuyoshi@me.com

The corresponding authors share this e-mail address.

Supplementary Information

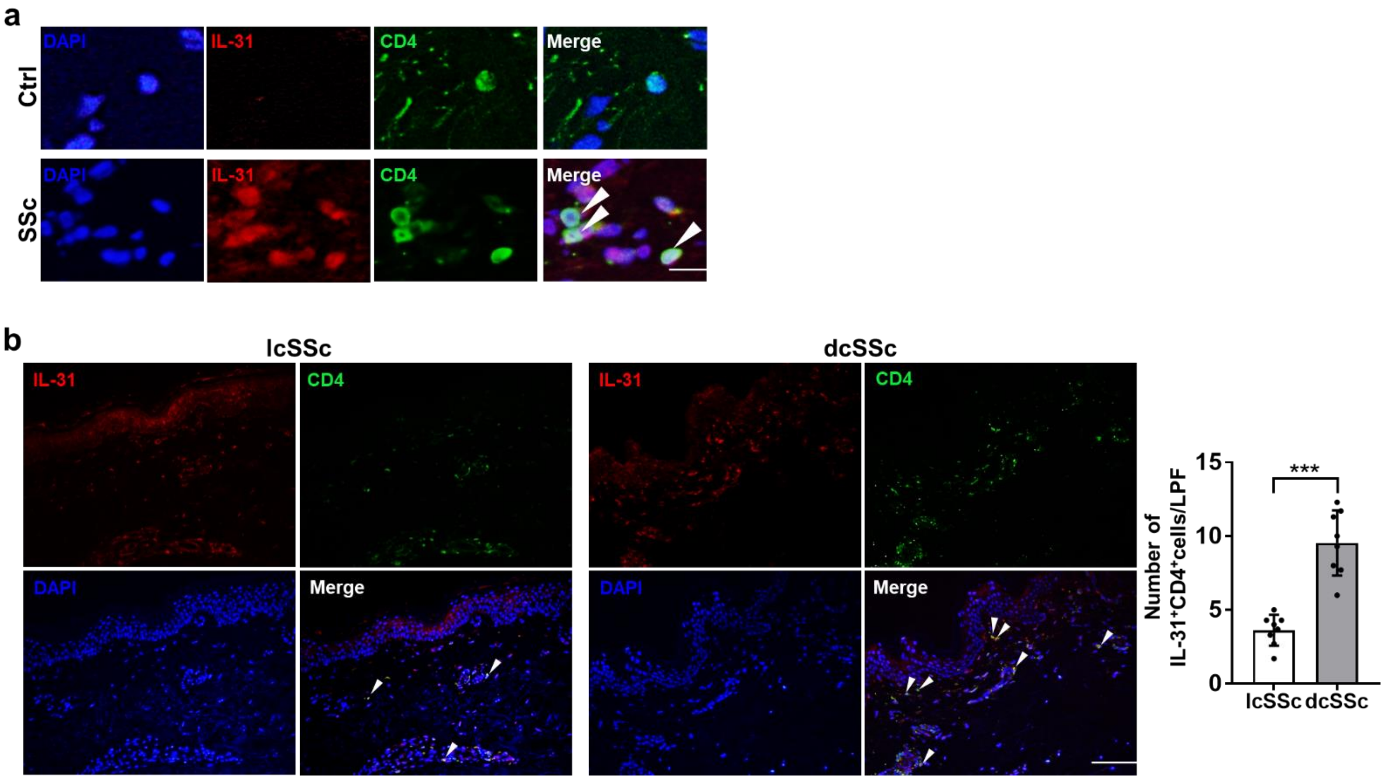

Supplementary Figure 1. IL-31 expression by CD4^{+} cells in human skin biopsies.

a. Representative double-immunofluorescence images for IL-31 (red), CD4 (green), and nuclei (DAPI, blue) in the skin biopsies from healthy controls and SSc patients (horizontal scale bars=10 μm). The results shown are representative of three independent experiments with similar results. b. (Left) Representative double-immunofluorescence images for IL-31 (red), CD4 (green), and nuclei (DAPI, blue) in the skin biopsies from dcSSc and lcSSc patients (horizontal scale bars=100 μm). (Right) The number of CD4^{+}IL-31^{+} cells were compared between the skin biopsies from dcSSc and lcSSc patients (n=8, respectively). Arrow heads show CD4^{+}IL-31^{+} cells. Exact p value = 0.0002. Data are shown as mean ± SD. ***p<0.001 by two-tailed Mann-Whitney *U* test. Ctrl, healthy controls; LPF, low power field. Source data are provided as a Source Data file.

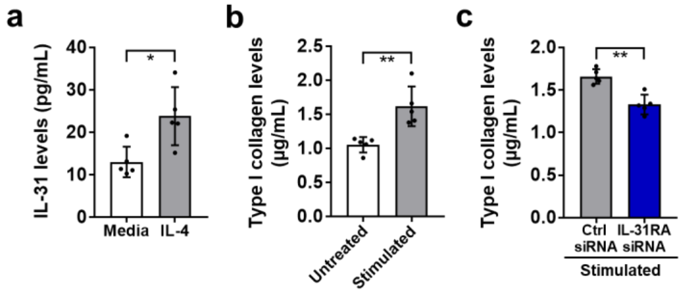

Suppelementary Figure 2. Collagen release was enhanced by the conditioned media of SSc DFs via IL-31.

a. IL-31 levels in the supernatants of SSc DFs stimulated with rhIL-4 (10 ng/mL) were evaluated by ELISA. Exact p value = 0.016. b. SSc DFs were stimulated with the conditioned media of other SSc DFs that were stimulated with rhIL-4 (10 ng/mL). Subsequently, type I collagen levels in the supernatants of the stimulated SSc DFs were measured by ELISA. Exact p value = 0.008. c. SSc DFs were pretreated with IL-31RA siRNA and stimulated with the conditioned media of SSc DFs that were stimulated with rhIL-4 (10 ng/mL). Subsequently, type I collagen levels in the supernatants of the stimulated SSc DFs were measured by ELISA. Exact p value = 0.008. n=5 biologically independent experiments. Data are shown as mean ± SD. *p<0.05 and **p<0.01 by two-tailed Mann-Whitney *U* test. Ctrl, control. Source data are provided as a Source Data file.

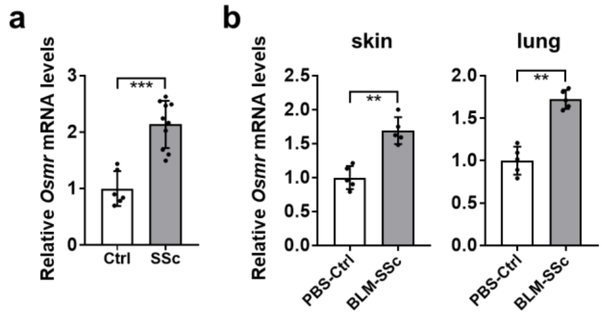

Supplementary Figure 3. OSMR expression in SSc patients and BLM-SSc mice.

a. Relative mRNA levels of *Osmr* were measured by real-time PCR in the skin biopsies from healthy control (n=6) and SSc patients (n=10). Exact p value = 0.0002. Relative fold difference = 2.14. b. Relative mRNA levels of *Osmr* were measured by real-time PCR in the skin and lungs of PBS-Ctrl and BLM-SSc mice (n=5, respectively). Exact p values = 0.008 (skin); 0.008 (lung). Relative fold differences = 1.69 (skin); 1.72 (lung). Data are shown as mean ± SD. $^{**}p<0.01$ and $^{***}p<0.001$ by two-tailed Mann-Whitney *U* test. Ctrl, healthy controls; PBS-Ctrl, PBS-treated control. Source data are provided as a Source Data file.

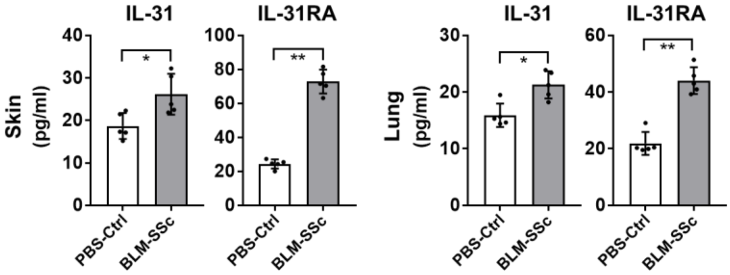

Supplementary Figure 4. IL-31 and IL-31RA expression in BLM-SSc mice.

Protein levels of IL-31 and IL-31RA were assessed by ELISA in the skin and lungs of PBS-Ctrl and BLM-SSc mice. Exact p values = 0.016 (IL-31, skin); 0.008 (IL-31RA, skin); 0.016 (IL-31, lung); 0.008 (IL-31RA, lung). *$p<0.05$ and **$p<0.01$ by two-tailed Mann-Whitney *U* test. n=5. Data are shown as mean ± SD. PBS-Ctrl, PBS-treated control. Source data are provided as a Source Data file.

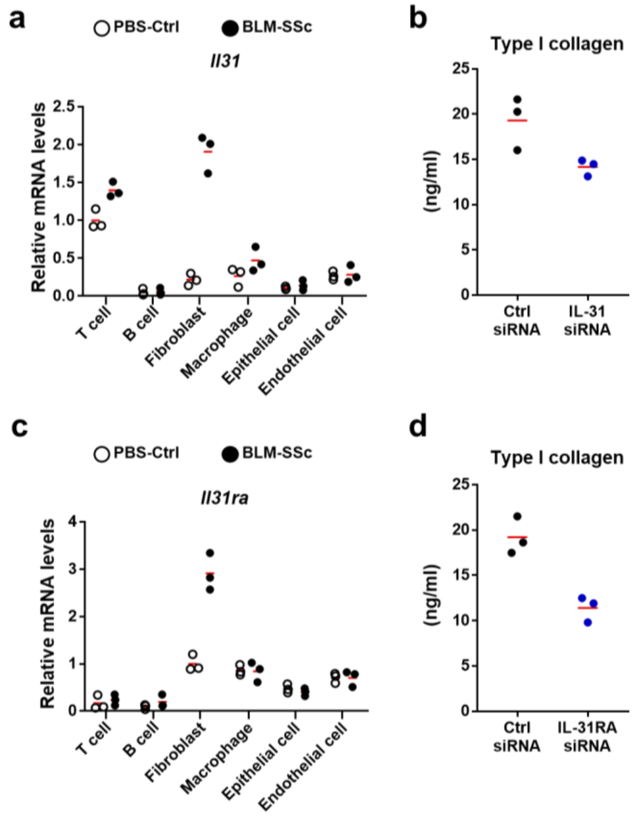

Supplementary Figure 5. IL-31 was primarily expressed by fibroblasts in the airways of BLM-SSc mice.

a, c. *Il31* (a) and *Il31ra* (c) expression was assessed by real time-PCR in T cells, B cells, fibroblasts, macrophages, epithelial cells, and endothelial cells that were isolated from the lungs of PBS-Ctrl and BLM-SSc mice. *Il31*; relative fold differences = 1.00 (T cell, PBS-Ctrl), 1.40 (T cell, BLM-SSc), 0.05 (B cell, PBS-Ctrl), 0.06 (B cell, BLM-SSc), 0.22 (fibroblast, PBS-Ctrl), 1.91 (fibroblast, BLM-SSc), 0.26 (macrophage, PBS-Ctrl), 0.47 (macrophage, BLM-SSc), 0.11 (epithelial cell, PBS-Ctrl), 0.14 (epithelial cell, BLM-SSc), 0.27 (endothelial cell, PBS-Ctrl), 0.28 (endothelial cell, BLM-SSc). *Il31ra*; relative fold differences = 0.17 (T cell, PBS-Ctrl), 0.24 (T cell, BLM-SSc), 0.09 (B cell, PBS-Ctrl), 0.19 (B cell, BLM-SSc), 1.00 (fibroblast, PBS-Ctrl), 2.91 (fibroblast, BLM-SSc), 0.86 (macrophage, PBS-Ctrl), 0.84 (macrophage, BLM-SSc), 0.47 (epithelial cell, PBS-Ctrl), 0.40 (epithelial cell, BLM-SSc), 0.71 (endothelial cell, PBS-Ctrl), 0.70 (endothelial cell, BLM-SSc). b, d. Fibroblasts from the lungs of BLM-SSc mice were treated with IL-31 siRNA (b) or IL-31RA siRNA (d) and the expression of type I collagen was evaluated by ELISA. Fibroblasts treated with control siRNA were used as controls. n=3. Red lines show mean values. PBS-Ctrl, PBS-treated control; Ctrl, control. Source data are provided as a Source Data file.

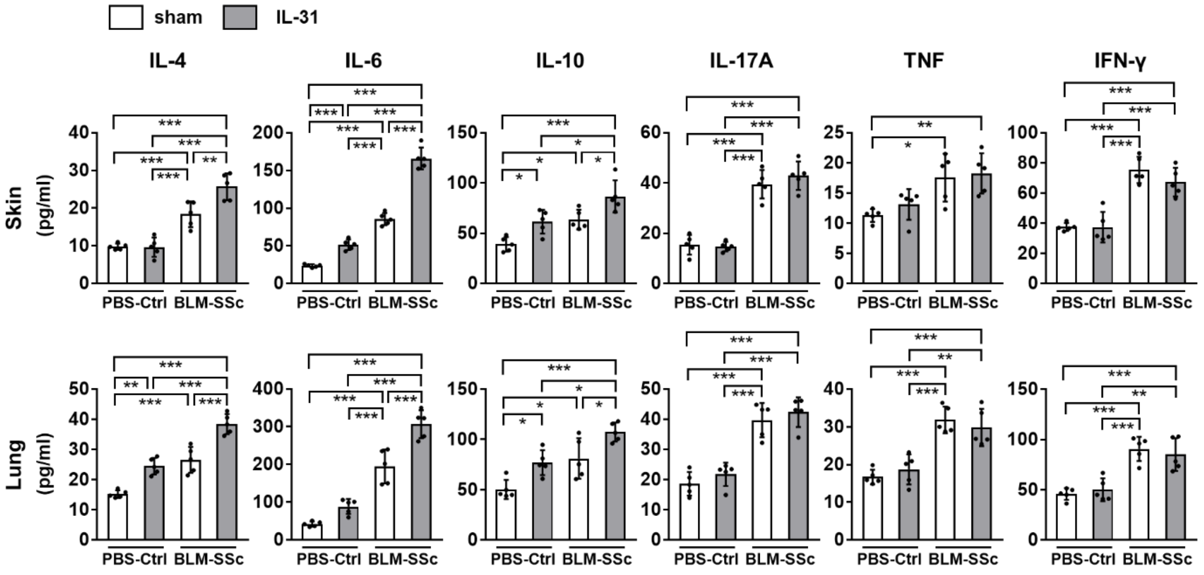

Supplementary Figure 6. Cytokine expression levels in PBS-Ctrl and BLM-SSc mice.

Protein levels of IL-4, IL-6, IL-10, IL-17A, TNF, and IFN-γ were assessed by ELISA in the skin and lungs of PBS-Ctrl and BLM-SSc mice treated with IL-31 or sham. Exact p values (PBS-Ctrl + sham vs. PBS-Ctrl + IL-31, PBS-Ctrl + sham vs. BLM-SSc + sham, PBS-Ctrl + sham vs. BLM-SSc + IL-31, PBS-Ctrl + IL-31 vs. BLM-SSc + sham, PBS-Ctrl + IL-31 vs. BLM-SSc + IL-31, BLM-SSc + sham vs. BLM-SSc + IL-31) = 0.999, 0.0009, 0.0000007, 0.0008, 0.0000006, 0.004 (IL-4, skin); 0.001, 0.00000006, 0.0000000000003, 0.0001, 0.000000000006, 0.000000001 (IL-6, skin); 0.034, 0.020, 0.00004, 0.991, 0.017, 0.030 (IL-10, skin); 0.994, 0.000002, 0.0000003, 0.000001, 0.0000002, 0.648 (IL-17A, skin); 0.761, 0.018, 0.008, 0.117, 0.059, 0.982 (TNF, skin); 0.999, 0.00001, 0.0002, 0.00001, 0.0002, 0.433 (IFN-γ, skin); 0.001, 0.0002, 0.00000002, 0.729, 0.00001, 0.0001 (IL-4, lung); 0.123, 0.000004, 0.000000002, 0.0003, 0.00000003, 0.0002 (IL-6, lung); 0.032, 0.012, 0.00003, 0.960, 0.013, 0.035 (IL-10, lung); 0.715, 0.00001, 0.000003, 0.00009, 0.00002, 0.800 (IL-17A, lung); 0.833, 0.00005, 0.0003, 0.0003, 0.001, 0.837 (TNF, lung); 0.942, 0.0001, 0.0005, 0.0004, 0.002, 0.887 (IFN-γ, lung). n=5. Data are shown as mean ± SD. *p<0.05, **p<0.01, and ***p<0.001. One-way analysis of variance followed by Tukey's post hoc comparison test was used. The results shown are representative of three independent experiments with similar results. PBS-Ctrl, PBS-treated control. Source data are provided as a Source Data file.

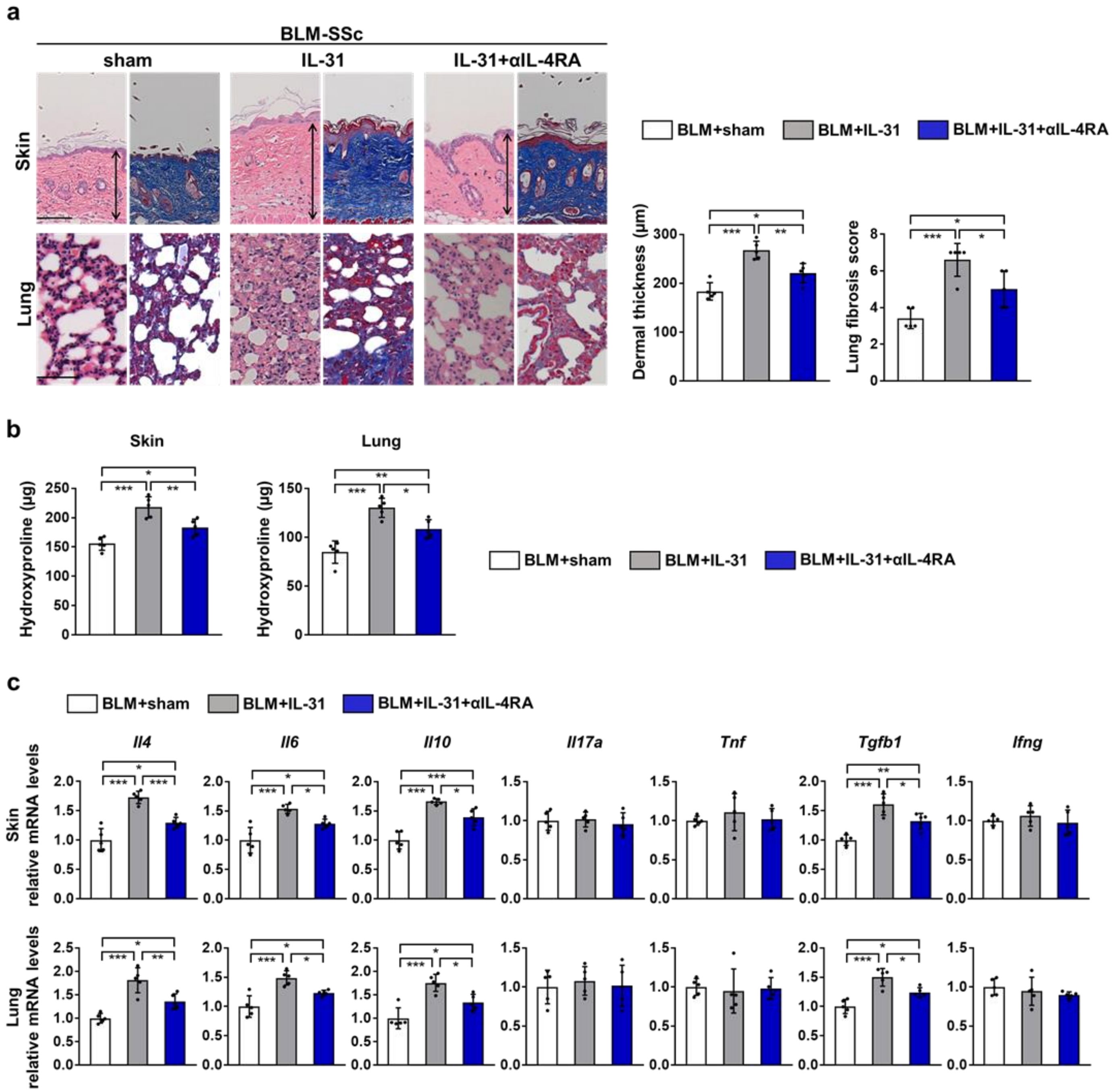

Supplementary Figure 7. Anti-IL-4RA mAb ameliorated fibrosis and Th2 polarization induced by IL-31 in BLM-SSc mice.

Mice were administered with either BLM and sham (day 1-14, respectively), BLM and rmIL-31 (day 1-14, respectively), or BLM (day 1-14), rmIL-31 (day 1-14), and anti-IL-4RA mAb (day 1, 4, 8, 11), and analyzed on day 15. Saline was used as a sham for rmIL-31. n=5. a. Representative histological sections stained with hematoxylin and eosin (left) and Masson trichrome (right) of the skin and lungs were shown (horizontal scale bars=100 μm in skin; 20 μm in lung). Vertical bars with arrows represent dermal thickness. Dermal thickness and lung fibrosis score were assessed histologically. Exact p values (BLM-SSc + sham vs. BLM-SSc + IL-31, BLM-SSc + sham vs. BLM-SSc + IL-31 + αIL-4RA, BLM-SSc + IL-31 vs. BLM-SSc + IL-31 + αIL-4RA) = 0.00004, 0.022, 0.005 (dermal thickness); 0.0002, 0.027, 0.027 (lung fibrosis score). b. Hydroxyproline contents of skin and lung samples. Exact p values (BLM-SSc + sham vs. BLM-SSc + IL-31, BLM-SSc + sham vs. BLM-SSc + IL-31 + αIL-4RA, BLM-SSc + IL-31 vs. BLM-SSc + IL-31 + αIL-4RA) = 0.00006, 0.029, 0.008 (skin); 0.00004, 0.008, 0.016 (lung). c. Relative mRNA expression levels of *Il4*, *Il6*, *Il10*, *Il17a*, *Tnf*, *Tgfb1*, and *Ifng* in the skin and lungs were evaluated by real-time PCR. Exact p values (BLM-SSc + sham vs. BLM-SSc + IL-31, BLM-SSc + sham vs. BLM-

SSc + IL-31 + αIL-4RA, BLM-SSc + IL-31 vs. BLM-SSc + IL-31 + αIL-4RA) = 0.000007, 0.014, 0.001 (*Il4*, skin); 0.0002, 0.022, 0.039 (*Il6*, skin); 0.000006, 0.0009, 0.013 (*Il10*, skin); 0.975, 0.850, 0.733 (*Il17a*, skin); 0.554, 0.984, 0.656 (*Tnf*, skin); 0.00004, 0.008, 0.018 (*Tgfb1*, skin); 0.723, 0.950, 0.542 (*Ifng*, skin); 0.00006, 0.029, 0.007 (*Il4*, lung); 0.0002, 0.039, 0.022 (*Il6*, lung); 0.0001, 0.043, 0.013 (*Il10*, lung); 0.844, 0.993, 0.896 (*Il17a*, lung); 0.906, 0.989, 0.957 (*Tnf*, lung); 0.00008, 0.022, 0.014 (*Tgfb1*, lung); 0.771, 0.401, 0.800 (*Ifng*, lung). Relative fold differences (BLM + IL-31, BLM + IL-31 + αIL-4RA) = 1.73, 1.30 (*Il4*, skin); 1.54, 1.28 (*Il6*, skin); 1.66, 1.39 (*Il10*, skin); 1.02, 0.96 (*Il17a*, skin); 1.11, 1.02 (*Tnf*, skin); 1.61, 1.33 (*Tgfb1*, skin); 1.06, 0.98 (*Ifng*, skin); 1.81, 1.36 (*Il4*, lung); 1.49, 1.23 (*Il6*, lung); 1.76, 1.34 (*Il10*, lung); 1.08, 1.02 (*Il17a*, lung); 0.95, 0.98 (*Tnf*, lung); 1.50, 1.24 (*Tgfb1*, lung); 0.95, 0.90 (*Ifng*, lung). Data are shown as mean ± SD. *p<0.05, **p<0.01, and ***p<0.001. One-way analysis of variance followed by Tukey's post hoc comparison test was used. The results shown are representative of three independent experiments with similar results. αIL-4RA, anti-IL-4RA mAb. Source data are provided as a Source Data file.

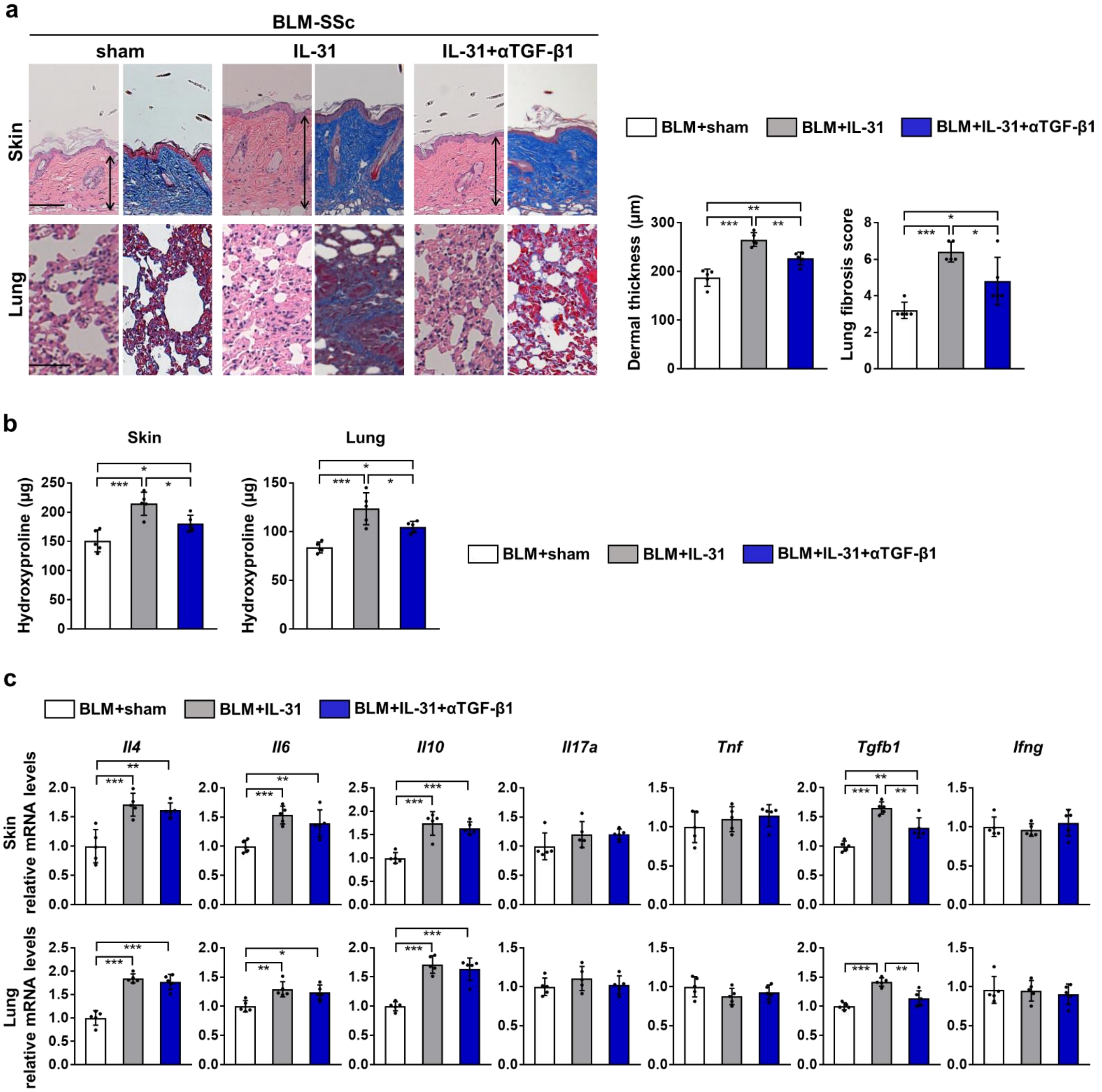

Supplementary Figure 8. Anti-TGF-β1 mAb ameliorated fibrosis but not Th2 polarization induced by IL-31 in BLM-SSc mice.

Mice were administered with either BLM and sham (day 1-14, respectively), BLM and rmIL-31 (day 1-14, respectively), or BLM (day 1-14), rmIL-31 (day 1-14), and anti-TGF-β1 mAb (day 1, 4, 8, 11), and analyzed on day 15. Saline was used as a sham for rmIL-31. n=5. a. Representative histological sections stained with hematoxylin and eosin (left) and Masson trichrome (right) of the skin and lungs were shown (horizontal scale bars=100 μm in skin; 20 μm in lung). Vertical bars with arrows represent dermal thickness. Dermal thickness and lung fibrosis score were assessed histologically. Exact p values (BLM-SSc + sham vs. BLM-SSc + IL-31, BLM-SSc + sham vs. BLM-SSc + IL-31 + αTGF-β1, BLM-SSc + IL-31 vs. BLM-SSc + IL-31 + αTGF-β1) = 0.000008, 0.004, 0.004 (skin); 0.0002, 0.030, 0.030 (lung). b. Hydroxyproline contents of skin and lung samples. Exact p values (BLM-SSc + sham vs. BLM-SSc + IL-31, BLM-SSc + sham vs. BLM-SSc + IL-31 + αTGF-β1, BLM-SSc + IL-31 vs. BLM-SSc + IL-31 + αTGF-β1) = 0.0003, 0.049, 0.027 (skin); 0.0002, 0.021, 0.038 (lung). c. Relative mRNA expression levels of *Il4*, *Il6*, *Il10*, *Il17a*, *Tnf*, *Tgfb1*, and *Ifng* in the skin and lungs were evaluated by real-

time PCR. Exact p values (BLM-SSc + sham vs. BLM-SSc + IL-31, BLM-SSc + sham vs. BLM-SSc + IL-31 + αTGF-β1, BLM-SSc + IL-31 vs. BLM-SSc + IL-31 + αTGF-β1) = 0.0005, 0.002, 0.763 (*Il4*, skin); 0.0009, 0.009, 0.415 (*Il6*, skin); 0.00007, 0.0003, 0.646 (*Il10*, skin); 0.260, 0.238, 0.998 (*Il17a*, skin); 0.632, 0.390, 0.903 (*Tnf*, skin); 0.000008, 0.005, 0.003 (*Tgfb1*, skin); 0.874, 0.807, 0.517 (*Ifng*, skin); 0.000002, 0.000005, 0.680 (*Il4*, lung); 0.006, 0.020, 0.771 (*Il6*, lung); 0.00002, 0.00005, 0.684 (*Il10*, lung); 0.413, 0.953, 0.579 (*Il17a*, lung); 0.217, 0.576, 0.735 (*Tnf*, lung); 0.00004, 0.097, 0.001 (*Tgfb1*, lung); 0.993, 0.826, 0.884 (*Ifng*, lung). Relative fold differences (BLM-SSc + IL-31, BLM-SSc + IL-31 + αTGF-β1) = 1.71, 1.62 (*Il4*, skin); 1.54, 1.39 (*Il6*, skin); 1.74, 1.64 (*Il10*, skin); 1.20, 1.21 (*Il17a*, skin); 1.10, 1.15 (*Tnf*, skin); 1.65, 1.32 (*Tgfb1*, skin); 0.96, 1.05 (*Ifng*, skin); 1.85, 1.77 (*Il4*, lung); 1.29, 1.24 (*Il6*, lung); 1.71, 1.63 (*Il10*, lung); 1.11, 1.02 (*Il17a*, lung); 0.88, 0.93 (*Tnf*, lung); 1.41, 1.14 (*Tgfb1*, lung); 0.95, 0.91 (*Ifng*, lung). Data are shown as mean ± SD. *$p<0.05$, **$p<0.01$, and ***$p<0.001$. One-way analysis of variance followed by Tukey's post hoc comparison test was used. The results shown are representative of three independent experiments with similar results. αTGF-β1, anti-TGF-β1 mAb. Source data are provided as a Source Data file.

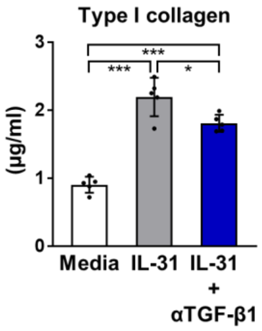

Supplementary Figure 9. Anti-TGF-β1 antibody diminished the collagen induction by IL-31 in SSc DFs.

Fibroblasts from the skin of SSc patients were treated with media alone, IL-31 (50 ng/ml), or IL-31 and anti-TGF-β1 antibody (5 µg/ml), and the expression of type I collagen was evaluated by ELISA. Exact p values (media vs. IL-31, media vs. IL-31 + αTGF-β1, IL-31 vs. IL-31 + αTGF-β1) = 0.0000005, 0.00002, 0.019. Data are shown as mean ± SD. *$p<0.05$ and ***$p<0.001$. One-way analysis of variance followed by Tukey's post hoc comparison test was used. The results shown are representative of three independent experiments with similar results. n=5. αTGF-β1, anti-TGF-β1 antibody. Source data are provided as a Source Data file.

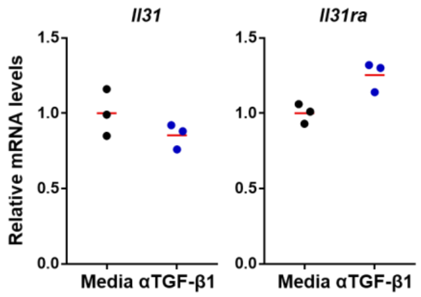

Supplementary Figure 10. Anti-TGF-β1 treatment decreased IL-31 expression and increased IL-31RA expression in fibroblasts from the lungs of BLM-SSc mice.

Fibroblasts from the lungs of BLM-SSc mice were treated with anti-TGF-β1 antibody (10 μg/ml) or media alone, and the expression of *Il31* and *Il31ra* was evaluated by real-time PCR. Relative fold differences = 0.85 (*Il31*); 1.25 (*Il31ra*). n=3. αTGF-β1, anti-TGF-β1 antibody. Source data are provided as a Source Data file.

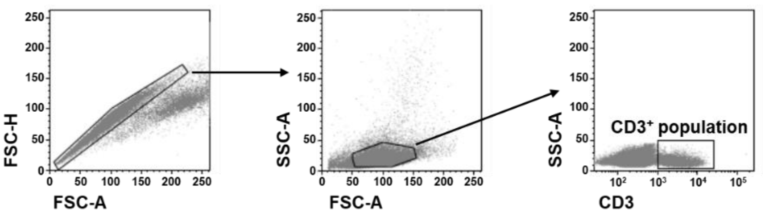

Supplementary Figure 11. Gating strategies used for flow cytometry analysis.

Gating strategy to analyze the frequencies of Th1, Th2, Th17, and Treg cells in spleen, lung, and lung-draining lymph nodes of BLM-SSc and PBS-treated control mice presented in Figures 7a and 9a. FSC-A, forward scatter area; FSC-H, forward scatter height; SSC-A, side scatter area.

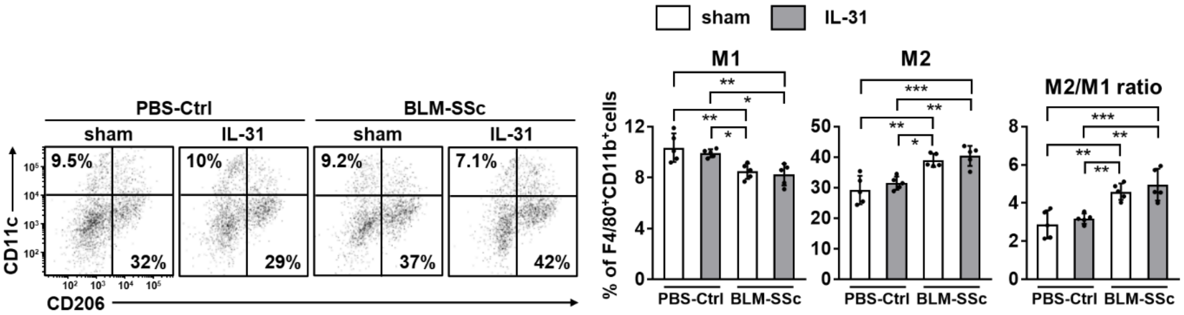

Supplementary Figure 12. IL-31 did not affect M1 and M2 macrophage differentiation.

Differentiation of M1 and M2 macrophages in the spleen was assessed by flow cytometry (n=5). (Left) Representative flow cytometry dot plots of CD11c and CD206 expression in $F4/80^+CD11b^+$ cells. (Right) M1 and M2 macrophages were defined as $F4/80^+CD11b^+CD11c^+$ $CD206^-$cells and $F4/80^+CD11b^+CD11c^-CD206^+$ cells, respectively. Data are shown as mean ± SD. Exact p values (PBS-Ctrl + sham vs. PBS-Ctrl + IL-31, PBS-Ctrl + sham vs. BLM-SSc + sham, PBS-Ctrl + sham vs. BLM-SSc + IL-31, PBS-Ctrl + IL-31 vs. BLM-SSc + sham, PBS-Ctrl + IL-31 vs. BLM-SSc + IL-31, BLM-SSc + sham vs. BLM-SSc + IL-31) = 0.811, 0.009, 0.003, 0.050, 0.018, 0.952 (M1); 0.683, 0.001, 0.0003, 0.011, 0.003, 0.893 (M2); 0.863, 0.002, 0.0003, 0.010, 0.002, 0.784 (M2/M2 ratio). *p<0.05, **p<0.01, and ***p<0.001. One-way analysis of variance followed by Tukey's post hoc comparison test was used. The results shown are representative of three independent experiments with similar results. PBS-Ctrl, PBS-treated control. Source data are provided as a Source Data file.

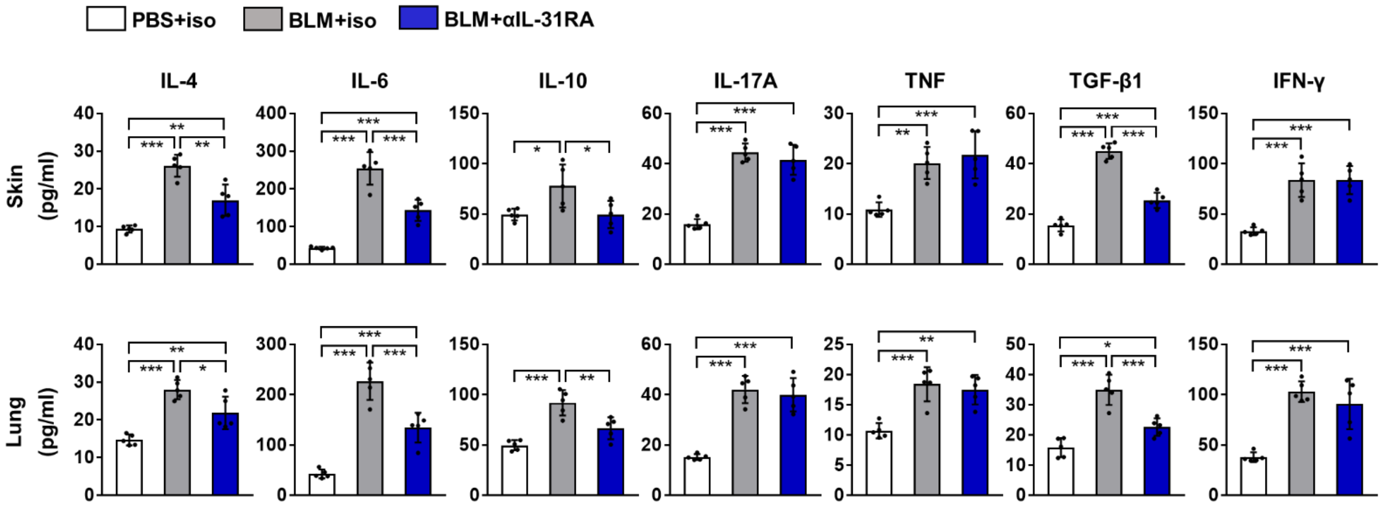

Supplementary Figure 13. Anti-IL-31RA mAb attenuated the protein expression of the cytokines associated with fibrosis and Th2 responses.

Protein expression levels of IL-4, IL-6, IL-10, IL-17A, TNF, TGF-β1, and IFN-γ in the skin and lungs were evaluated by ELISA (n=5). Data are shown as mean ± SD. *p<0.05, **p<0.01, and ***p<0.001. One-way analysis of variance followed by Tukey's post hoc comparison test was used. Exact p values (PBS-Ctrl + iso vs. BLM-SSc + iso, PBS-Ctrl + iso vs. BLM-SSc + αIL-31RA, BLM-SSc + iso vs. BLM-SSc + αIL-31RA) = 0.000003, 0.004, 0.001 (IL-4, skin); 0.0000003, 0.0005, 0..0002 (IL-6, skin); 0.028, 0.999, 0.029 (IL-10, skin); 0.0000005, 0.000002, 0.550 (IL-17A, skin); 0.003, 0.0007, 0.736 (TNF, skin); 0.000000004, 0.0004, 0.0000004 (TGF-β1, skin); 0.0001, 0.0001, 0.999 (IFN-γ, skin); 0.00004, 0.007, 0.018 (IL-4, lung); 0.0000006, 0.0006, 0.0005 (IL-6, lung); 0.00007, 0.051, 0.005 (IL-10, lung); 0.000005, 0.00001, 0.783 (IL-17A, lung); 0.0005, 0.001, 0.805 (TNF, lung); 0.00001, 0.038, 0.0007 (TGF-β1, lung); 0.00008, 0.0005, 0.464 (IFN-γ, lung). The results shown are representative of three independent experiments with similar results. αIL-31RA, anti-IL-31RA mAb. Source data are provided as a Source Data file.

Supplementary Table 1. Sequences of the primers used for real-time PCR.

Gene	Forward	Reverse
Human		
Il31	5' -GATGATGTACAGAAAATAGTCGAGGAATT- 3′	5' -CTTCTCTTCCTCCACATCTTTCAAA- 3′
Il31ra	5' -GGCATGGAGATGATTTCAAGGATAAGCTAAACCTG- 3′	5' -CTGGCTTCATCTGTGAAAATTTCTTGCAGAAC- 3′
Col1a1	5' -CCAGAAGAACTGGTACATCAGCA- 3′	5' -CGCCATACTCGAACTGGGAAT- 3′
Col1a2	5' -GATGTTGAACTTGTTGCTGAGG- 3′	5' -TCTTTCCCCATTCATTTGTCTT- 3′
Acta2	5' -TCTGGAGATGGTGTCACCCACAAT- 3′	5' -AATAGCCACGCTCAGTCAGG- 3′
Il6	5' -TAATGGGCATTCCTTCTTCT- 3′	5' -TGTCCTAACGCTCATACTTTT- 3′
Il33	5' -GGAAGAACACAGCAAGCAAAGCCT- 3′	5' -TAAGGCCAGAGCGGAGCTTCATAA- 3′
Ccl2	5' -AACCACAGTTCTACCCCTGGG- 3′	5' -TAATGATTCTTGCAAAGACCCTCAA- 3′
Osmr	5' -ATGGCTCTATTTGCAGTCTTTCA- 3′	5' -CACCCAGATGACATTGGATGTT- 3′
Tgfb1	5' -GGACATCAACGGGTTCACTA- 3′	5' -GCCATGAGAAGCAGGAAAG- 3′
Ctgf	5' -TTGCGAAGCTGACCTGGAAGAGAA- 3′	5' -AGCTCGGTATGTCTTCATGCTGGT- 3′
Mmp1	5' -GCTAACCTTTGATGCTATAACTACGA- 3′	5' -GGATTTGTGCGCATGTAGAA- 3′
Mmp3	5' -GTACCAACCTATTCCTGGTTGC- 3′	5' -CCAGAGAGTTAGATTTGGTGGG- 3′
Mmp9	5′ -ATTTCTGCCAGGACCGCTTCTACT- 3′	5' -CAGTTTGTATCCGGCAAACTGGCT- 3′
Gapdh	5' -ACCCACTCCTCCACCTTTGA- 3′	5' -CATACCAGGAAATGAGCTTGACAA- 3′
Mouse		
Il31	5' -TTCCACACAGGAACAACGAA- 3′	5' -TGATTCGTCTGCTGACATCC- 3′
Il31ra	5' -CCAGAAGCTGCCATGTCGAA- 3′	5' -TCTCCAACTCGGTGTCCCAAC- 3′
Il4	5' -CAACGAAGAACACCACAGAG- 3′	5' -GGACTTGGACTCATTCATGG- 3′
Il6	5' -GATGGATGCTACCAAACTGGAT- 3′	5' -CCAGGTAGCTATGGTACTCCAGA- 3′
Il10	5' -TTTGAATTCCCTGGGTGAGAA- 3′	5' -ACAGGGGAGAAATCGATGACA- 3′
Il17a	5' -CAGCAGCGATCATCCCTCAAAG- 3′	5' -CAGGACCAGGATCTCTTGCTG- 3′
Tnf	5' -ACCCTCACACTCAGATCATCTTC- 3′	5' -TGGTGGTTTGCTACGACGT- 3′
Tgfb1	5' -GCAACATGTGGAACTCTACCAGAA- 3′	5' -GACGTCAAAAGACAGCCACTCA- 3′
Ifng	5' -TCAAGTGGCATAGATGTGGAAGAA- 3′	5' -TGGCTCTGCAGGATTTTCATG- 3′
Ctgf	5' -GTGCCAGAACGCACACTG- 3'	5' -CCCCGGTTACACTCCAAA- 3'
Col1a1	5' -TCCCTCAGCTACCAAACACA- 3′	5' -TGCAGAATGAACAGGTAGAAGG- 3′
Col1a2	5' -GCTGTGGAAGGGGTCATAAA- 3′	5' -CCCTCCTCCATCCTCTCTCT- 3′
Osmr	5' -AAACATGATATTTCAGATAGAGATCAGTAGACT- 3′	5' -CTTATGAAATGTTTGACACACTCCAA- 3′
Mmp3	5' -GGCTGTGTGTGGTTGTGTGCTC- 3′	5' -CCTCCTCCCAGACCTTCAAAGC- 3′
Mmp9	5' -TCAGGGAGATGCCCATTTCG- 3′	5' -GAACGGGAACACACAGGGTTTG- 3′
Mmp13	5' -TTCTGAGGCCTTCAAGGAAA- 3′	5' -ATAGTGGGGAGAAGCAGCAG- 3′
Timp1	5' -ACAAGTCCCAGAACCGCAGTGA- 3′	5' -CCGTCCACAAACAGTGAGTGTCA- 3′
Timp2	5' -GAGCCACCGCAGTGAGCGAGAA- 3′	5' -GGGGAGGAGATGTAGCAAGGGA- 3′
Timp3	5' -CACGGAAGCCTCTGAAAGTC- 3′	5' -TCCACAAAGTTGCACAGTCC- 3′
Gapdh	5' -CGTGTTCCTACCCCCAATGT- 3′	5' -TGTCATCATACTTGGCAGGTTTCT- 3′

Supplementary Table 2. Details of the antibodies used.

Target	Reactivity	Conjugate	Clone	Source	Catalog#	dilution
IL-31	Human	None	polyclonal	Abcam	ab102750	1/125
IL-31RA	Human	None	polyclonal	Abcam	ab113498	1/250
STAT1	Human	None	polyclonal	Cell Signaling Technology	9172	1/1000
STAT3	Human	None	79D7	Cell Signaling Technology	4904	1/2000
STAT5	Human	None	D2O6Y	Cell Signaling Technology	94205	1/1000
pSTAT1	Human	None	D4A7	Cell Signaling Technology	7649	1/1000
pSTAT3	Human	None	D3A7	Cell Signaling Technology	9145	1/2000
pSTAT5	Human	None	D47E7	Cell Signaling Technology	4322	1/1000
α-SMA	Human	None	D4K9N	Cell Signaling Technology	19245	1/1000
FSP-1	Human	None	polyclonal	GeneTex	GTX32855	1/100
CD4	Human	None	4B12	Invitrogen	MA5-12259	1/20
CD3	Mouse	PE	145-2C11	eBioscience	12-0031	1/200
CD4	Mouse	FITC	GK1.5	eBioscience	11-0041	1/100
IFN-γ	Mouse	APC	XMG1.2	eBioscience	17-7311	1/200
IL-4	Mouse	APC	11B11	eBioscience	17-7041	1/200
IL-17A	Mouse	APC	eBio17B7	eBioscience	11-7177	1/200
CD3	Mouse	PE/Cy7	145-2C11	eBioscience	25-0031	1/200
CD25	Mouse	APC	PC61.5	eBioscience	17-0251	1/200
Foxp3	Mouse	PE	FJK-16s	eBioscience	12-5773	1/200
F4/80	Mouse	FITC	BM8	eBioscience	11-4801	1/100
CD11b	Mouse	APC	M1/70	eBioscience	17-0112	1/200
CD206	Mouse	PE	MR6F3	eBioscience	12-2061	1/200
CD11c	Mouse	PE/Cy7	N418	eBioscience	25-0114	1/200

Uncropped images

Figure 3d

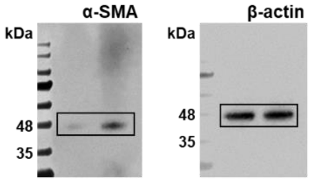

Figure 4a

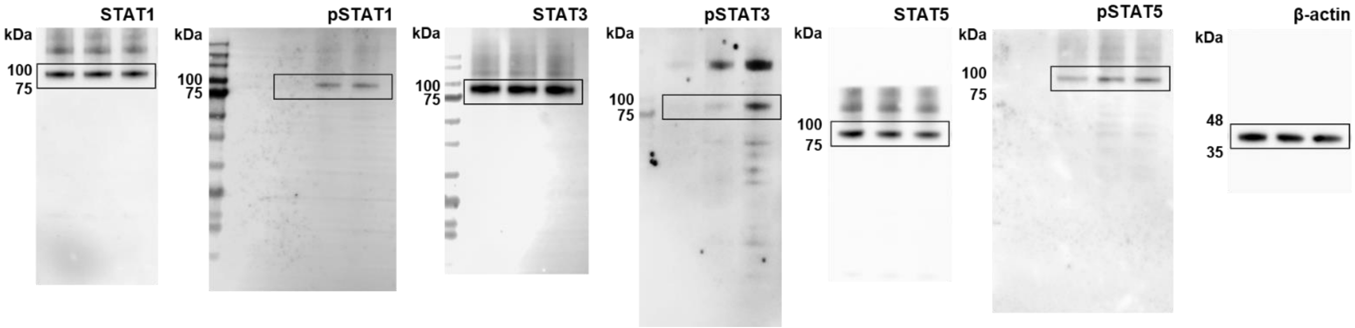

Supplement: Supplementary file 1 — Supplementary Information [file 41467_2021_26099_MOESM1_ESM.pdf]
