# Supplementary material for: Interleukin-31 promotes fibrosis and T helper 2 polarization in systemic sclerosis
Source: Nat Commun. 2021 Oct 12;12:5947. doi: 10.1038/s41467-021-26099-w (PMC8511151; doi:10.1038/s41467-021-26099-w)
Supplement: Supplementary file 2 — Reporting Summary [file 41467_2021_26099_MOESM2_ESM.pdf]

nature research

Corresponding author(s): Ayumi Yoshizaki

Last updated by author(s): Jun 29, 2021

Reporting Summary

Nature Research wishes to improve the reproducibility of the work that we publish. This form provides structure for consistency and transparency in reporting. For further information on Nature Research policies, see our Editorial Policies and the Editorial Policy Checklist.

Statistics

For all statistical analyses, confirm that the following items are present in the figure legend, table legend, main text, or Methods section.

n/a	Confirmed	
☐	☒	The exact sample size (*n*) for each experimental group/condition, given as a discrete number and unit of measurement
☐	☒	A statement on whether measurements were taken from distinct samples or whether the same sample was measured repeatedly
☐	☒	The statistical test(s) used AND whether they are one- or two-sided<br>*Only common tests should be described solely by name; describe more complex techniques in the Methods section.*
☐	☒	A description of all covariates tested
☐	☒	A description of any assumptions or corrections, such as tests of normality and adjustment for multiple comparisons
☐	☒	A full description of the statistical parameters including central tendency (e.g. means) or other basic estimates (e.g. regression coefficient) AND variation (e.g. standard deviation) or associated estimates of uncertainty (e.g. confidence intervals)
☐	☒	For null hypothesis testing, the test statistic (e.g. *F*, *t*, *r*) with confidence intervals, effect sizes, degrees of freedom and *P* value noted<br>*Give P values as exact values whenever suitable.*
☒	☐	For Bayesian analysis, information on the choice of priors and Markov chain Monte Carlo settings
☒	☐	For hierarchical and complex designs, identification of the appropriate level for tests and full reporting of outcomes
☐	☒	Estimates of effect sizes (e.g. Cohen's *d*, Pearson's *r*), indicating how they were calculated

Our web collection on statistics for biologists contains articles on many of the points above.

Software and code

Policy information about availability of computer code

Data collection	FACSuite 1.0.5 3841 was used to collect the flow cytometry data.
Data analysis	-GraphPad Prism 7.03 was used to analyze all data.<br>-ImageJ 1.53e was used to normalize the western blot data.<br>-Kaluza 2.1 was used to analyze the flow cytometry data.

For manuscripts utilizing custom algorithms or software that are central to the research but not yet described in published literature, software must be made available to editors and reviewers. We strongly encourage code deposition in a community repository (e.g. GitHub). See the Nature Research guidelines for submitting code & software for further information.

Data

Policy information about availability of data

All manuscripts must include a data availability statement. This statement should provide the following information, where applicable:

- Accession codes, unique identifiers, or web links for publicly available datasets
- A list of figures that have associated raw data
- A description of any restrictions on data availability

All data is available in the article, supplementary information, or from the corresponding author upon reasonable request. Source images are available at Mendeley Data (doi: 10.17632/dhj6bmryy5.1). Source Data are provided with this paper.

Field-specific reporting

Please select the one below that is the best fit for your research. If you are not sure, read the appropriate sections before making your selection.

- [x] Life sciences
- [ ] Behavioural & social sciences
- [ ] Ecological, evolutionary & environmental sciences

For a reference copy of the document with all sections, see nature.com/documents/nr-reporting-summary-flat.pdf

Life sciences study design

All studies must disclose on these points even when the disclosure is negative.

Sample size	-Sample sizes were determined based on the previously published papers (ref:1-3)<br>-Reference:<br>1) Jeljeli M, et al., Nat Commun. 2019;10(1):5670.<br>2) Matsushita T, et al., Sci Adv. 2018;4(7):eaas9944.<br>3) Mor A, et al., Ann Rheum Dis. 2019;78(9):1260-1268.
Data exclusions	No data were excluded from the analyses.
Replication	In vivo experiments were performed three times with similar results (Figures 5-7, Supplementary Figures 6-8, 11, and 12).
Randomization	- All animals were grouped randomly.<br>- All in vitro samples were allocated randomly.
Blinding	Investigators were blinded to group allocation during data collection and analysis.

Reporting for specific materials, systems and methods

We require information from authors about some types of materials, experimental systems and methods used in many studies. Here, indicate whether each material, system or method listed is relevant to your study. If you are not sure if a list item applies to your research, read the appropriate section before selecting a response.

Materials & experimental systems

n/a	Involved in the study	
[ ]	[x]	Antibodies
[x]	[ ]	Eukaryotic cell lines
[x]	[ ]	Palaeontology and archaeology
[ ]	[x]	Animals and other organisms
[ ]	[x]	Human research participants
[x]	[ ]	Clinical data
[x]	[ ]	Dual use research of concern

Methods

n/a	Involved in the study	
[x]	[ ]	ChIP-seq
[ ]	[x]	Flow cytometry
[x]	[ ]	MRI-based neuroimaging

Antibodies

Antibodies used	Rabbit anti-IL-31 antibody (polyclonal, Catalog#ab102750, dilution 1/125), rabbit anti-IL-31RA antibody (polyclonal, Catalog#ab113498, dilution 1/250) from Abcam (Cambridge, MA, USA) and anti-IL-4RA antibody (mIL4R-M1, Catalog#551853) from BD Biosciences (San Diego, CA, USA) and STAT1 (polyclonal, Catalog#9172, dilution 1/1000), STAT3 (79D7, Catalog#4904, dilution 1/2000), STAT5 (D2O6Y, Catalog#94205, dilution 1/1000), phospho-STAT1 (D4A7, Catalog#7649, dilution 1/1000), phospho-STAT3 (D3A7, Catalog#9145, dilution 1/2000), phospho-STAT5 (D47E7, Catalog#4322, dilution 1/1000), α-SMA (D4K9N, Catalog#19245, dilution 1/1000), anti-mouse IgG-HRP (Catalog#7076, dilution 1/2000), anti-rabbit IgG-HRP (Catalog#7074, dilution 1/2000) from Cell Signaling Technology (Beverly, MA, USA) and mouse IgG1 kappa isotype control (P3.6.2.8.1, Catalog#14-4714), CD3-PE (145-2C11, Catalog#12-0031, dilution 1/200), CD4-FITC (GK1.5, Catalog#11-0041, dilution 1/100), IFN-γ-APC (XMG1.2, Catalog#17-7311, dilution 1/200), IL-4-APC (11B11, Catalog#17-7041, dilution 1/200), IL-17A-APC (eBio17B7, Catalog#11-7177, dilution 1/200), rat IgG1 kappa isotype control-APC (eBRG1, Catalog#17-4301, dilution 1/200), rat IgG2a kappa isotype control (eBR2a, Catalog#11-4321, dilution 1/200), CD3-PE/Cy7 (145-2C11, Catalog#25-0031, dilution 1/200), CD25-APC (PC61.5, Catalog#17-0251, dilution 1/200), Foxp3-PE (FJK-16s, Catalog#12-5773, dilution 1/200), rat IgG2a kappa isotype control-PE (eBR2a, Catalog#12-4321, dilution 1/200), F4/80-FITC (BM8, Catalog#11-4801, dilution 1/100), CD11b-APC (M1/70, Catalog#17-0112, dilution 1/200), CD206-PE (MR6F3, Catalog#12-2061, dilution 1/200), CD11c-PE/Cy7 (N418, Catalog#25-0114, dilution 1/200), donkey anti-rabbit IgG-Alexa Flour 594 (polyclonal, Catalog# A-11037, dilution 1/300), donkey anti-goat IgG-Alexa Fluor 488 (polyclonal, Catalog#A-11055, dilution 1/300), donkey anti-mouse IgG-Alexa Flour 488 (polyclonal, Catalog#A-21202, dilution 1/300) from eBioscience (San Diego, CA, USA) and goat anti-FSP-1 antibody (polyclonal, Catalog#GTX32855, dilution 1/100) from GeneTex (Irvine, CA, USA) and mouse anti-CD4 antibody (4B12, Catalog# MA5-12259, dilution 1/20) from Invitrogen (Carlsbad, CA, USA) and anti-human TGF-β1 antibody (1D11, Catalog#MAB2401, used at 1/100 dilution from 0.5 mg/ml stock solution), anti-mouse TGF-β1 antibody (1D11, Catalog#MAB1835, used at 1/50 dilution from 0.5 mg/ml stock solution) from R&D Systems (Minneapolis, MN, USA) and phospho-STAT3 (B-7, Catalog#sc-8059, dilution

	1/100), normal rabbit IgG (polyclonal, Catalog#sc-2025, dilution 1/200), β-actin (C4, Catalog#sc-47778, dilution 1/1000) from Santa Cruz Biotechnology (Santa Cruz, CA, USA) and PE-conjugated mouse anti-rabbit IgG mAb (2A9, Catalog#4090-09, dilution 1/100) from Southern Biotech (Birmingham, AL, USA).
Validation	-The neutralizing activity of anti-mouse IL-31RA antibody (NRA4900) was validated by its ability to inhibit IL-31-dependent proliferation of the Ba/F3 cells that were transfected with mouse IL-31RA and mouse OSMR genes.<br>-All other antibodies have been validated for the species and application by the manufacturers (Abcam, BD Biosciences, Cell Signaling Technology, eBioscience, GeneTex, Invitrogen, R&D Systems, Santa Cruz Biotechnology, Southern Biotech), which provide an extensive library of publications citing their products on the manufacturers' websites.

Animals and other organisms

Policy information about studies involving animals; ARRIVE guidelines recommended for reporting animal research

Laboratory animals	Wild-type C57BL/6 female mice were purchased from The Jackson Laboratory (Bar Harbor, ME, USA). All mice used in the study were six weeks old and housed under controlled temperature (21±1 ˚C) and relative humidity (60±10%) with a 12/12 h dark/light cycle.
Wild animals	The study did not involve wild animals.
Field-collected samples	The study did not involve samples from the field.
Ethics oversight	All studies involving animals were approved by the Committee on Animal Experimentation of the University of Tokyo Graduate School of Medicine.

Note that full information on the approval of the study protocol must also be provided in the manuscript.

Human research participants

Policy information about studies involving human research participants

Population characteristics	-Serum samples were obtained from 74 Japanese SSc patients (67 women and seven men; mean ± SD age, 51 ± 17 years; disease duration, 2.0 ± 2.2 years). All patients fulfilled the ACR/EULAR classification criteria for SSc. Patients treated with corticosteroids or other immunosuppressants prior to their first visits were excluded.<br>-As controls, 14 healthy Japanese individuals (13 women and one man; mean ± SD age, 51 ± 9.4 years) were enrolled.
Recruitment	-A total of 74 Japanese SSc patients who visited the University of Tokyo Hospital were recruited.<br>-A total of 14 Japanese individuals who visited the University of Tokyo Hospital for a routine checkup and had no past medical history were recruited as healthy controls.<br>-We tried to capture as many samples as possible without self-selection bias. We compared serum IL-31 levels between the two groups recruited at the same hospital, and the potential selection bias are unlikely to impact the results.
Ethics oversight	The whole study was approved by the ethics committee of the University of Tokyo Graduate School of Medicine. Written informed consent was obtained from all participants.

Note that full information on the approval of the study protocol must also be provided in the manuscript.

Flow Cytometry

Plots

Confirm that:

- [x] The axis labels state the marker and fluorochrome used (e.g. CD4-FITC).
- [x] The axis scales are clearly visible. Include numbers along axes only for bottom left plot of group (a 'group' is an analysis of identical markers).
- [ ] All plots are contour plots with outliers or pseudocolor plots.
- [x] A numerical value for number of cells or percentage (with statistics) is provided.

Methodology

Sample preparation	-Single cell suspensions from spleen and lung-draining lymph nodes were prepared by gentle teasing. Erythrocytes in spleen cell suspensions were subsequently lysed with ammonium chloride (#ST-07850, StemCell Technologies, Cambridge, MA, USA).<br>-Lung cell suspensions were prepared by incubating minced lung tissue in RPMI 1640 (#R8758, Sigma-Aldrich, St. Louis, MO, USA) with Liberase TM (#5401127001, Roche, Indianapolis, IN, USA, final 50 µg/ml) and DNase I (#10104159001,Roche, final 1 µg/ml) for 30 min, followed by the lysis of erythrocytes with ammonium chloride.
Instrument	Samples were analyzed with a FACS Verse flow cytometer (BD Biosciences, San Diego, CA, USA).
Software	Data were analyzed by Kaluza 2.1 (Beckmann Coulter, Miami, FL, USA).
Cell population abundance	No post-sort fractions were collected.

Gating strategy

-We applied forward and side scatter parameters (FSC, SSC) to exclude cell debris and doublets. Lymphocytes were identified as a population between 50 and 150 K of forward scatter and between 10 and 50 K of side scatter. Macrophages were identified as a population between 150 and 250 K of forward scatter and between 50 and 100 K of side scatter.

-Th1, Th2, and Th17 cells were identified as IFN-γ+, IL-4+, and IL-17A+ cells in CD3+CD4+ double positive population, respectively. Boundaries between positive and negative staining cell populations were defined at 10^3 of axis scale for CD3-PE, CD4-FITC, IFN-γ-APC, IL-4-APC, and IL-17A-APC.

-M1 and M2 macrophages were identified as F4/80+CD11b+CD11c+CD206-cells and F4/80+CD11b+CD11c-CD206+cells, respectively. Boundaries between positive and negative staining cell populations were defined at 10^4 of axis scale for CD11c-PE/Cy7, 10^3 of axis scale for F4/80-FITC, CD11b-APC, and 3.5x10^3 axis scale for CD206-PE.

☒ Tick this box to confirm that a figure exemplifying the gating strategy is provided in the Supplementary Information.
